# Phyto-microbiome to mitigate abiotic stress in crop plants

**DOI:** 10.3389/fmicb.2023.1210890

**Published:** 2023-08-02

**Authors:** Anamika Singh, Samina Mazahar, Shilpa Samir Chapadgaonkar, Priti Giri, Abhilasha Shourie

**Affiliations:** ^1^Department of Botany, Maitreyi College, University of Delhi, New Delhi, India; ^2^Department of Botany, Dyal Singh College, University of Delhi, New Delhi, India; ^3^Department of Biosciences and Technology, Dr. Vishwanath Karad MIT World Peace University, Pune, Maharashtra, India; ^4^Department of Biotechnology, Faculty of Engineering and Technology, Manav Rachna International Institute of Research and Studies, Faridabad, India

**Keywords:** phyto-microbiome, abiotic stress alleviation, bio-inoculants, multi-omics, plant microbiome engineering

## Abstract

Plant-associated microbes include taxonomically diverse communities of bacteria, archaebacteria, fungi, and viruses, which establish integral ecological relationships with the host plant and constitute the phyto-microbiome. The phyto-microbiome not only contributes in normal growth and development of plants but also plays a vital role in the maintenance of plant homeostasis during abiotic stress conditions. Owing to its immense metabolic potential, the phyto-microbiome provides the host plant with the capability to mitigate the abiotic stress through various mechanisms like production of antioxidants, plant growth hormones, bioactive compounds, detoxification of harmful chemicals and toxins, sequestration of reactive oxygen species and other free radicals. A deeper understanding of the structure and functions of the phyto-microbiome and the complex mechanisms of phyto-microbiome mediated abiotic stress mitigation would enable its utilization for abiotic stress alleviation of crop plants and development of stress-resistant crops. This review aims at exploring the potential of phyto-microbiome to alleviate drought, heat, salinity and heavy metal stress in crop plants and finding sustainable solutions to enhance the agricultural productivity. The mechanistic insights into the role of phytomicrobiome in imparting abiotic stress tolerance to plants have been summarized, that would be helpful in the development of novel bioinoculants. The high-throughput modern approaches involving candidate gene identification and target gene modification such as genomics, metagenomics, transcriptomics, metabolomics, and phyto-microbiome based genetic engineering have been discussed in wake of the ever-increasing demand of climate resilient crop plants.

## 1. Introduction

Climate change has lead to several perturbations in the environment such as extremes of heat and cold, drought, waterlogging, and changing weather patterns, which are responsible for adverse effects on crop production globally. Various environmental and anthropogenic factors pose abiotic stress on plants such as temperature, salinity, drought, heavy metals (Sandrini et al., 2022), UV radiation (Shourie et al., 2014), and pesticides (Yasmin and D’Souza, 2010). Temperature fluctuations, erratic rainfall and frequent droughts are also attributed to shifts in agricultural cycles. More than 50% of agricultural losses are caused due to heat, salinity, drought and heavy metal stresses, both qualitatively and quantitatively (Salam et al., 2023). It is worth noting that a minor temperature increase of even 1°C can reduce the crop yield of various crops by 3–7% (Zhao et al., 2017). These aspects are also responsible for changes in the edaphic factors like pH, moisture, salinity, ion content, mineral availability and organic content, which directly affect the crop yield. Salinity and heavy metal accumulation in soil has significant impacts on plant health and crop productivity. Salt stress inhibits seed germination and disturbs the homeostasis at cellular and biochemical level. It affects water uptake, exerts osmotic stress and causes nutritional imbalance in plants. Similarly, heavy metals also cause detrimental effects on plants by imposing toxicity and hampering physiological processes that are vital for survival and growth of plants.

Plants adopt various strategies to survive under unfavorable environmental conditions and have remarkable capabilities of enduring and adapting to abiotic stresses through transient and stable gene expression mediated by stress signaling. The microorganisms present in the soil, rhizosphere, and phyllosphere of plants play a crucial role in the maintenance of environmental homeostasis and enable plants to survive under stress conditions (Barea, 2015; Ngumbi and Kloepper, 2016). A plant acquires its microbiome from the parent plant, the soil in which the seed is sown, and the environment to which it is exposed. Plants and their microbiome are in an exquisite symbiotic relationship and mutually promote growth, health, and development. Plant-associated microbes offer numerous benefits to plants such as fixing atmospheric nitrogen, enhancing the bioavailability of minerals, producing organic nutrients, detoxifying pesticides, harmful chemicals and toxins, mitigating plant diseases, and producing plant growth hormones and bioactive compounds (Sagar et al., 2021). The rhizospheric occupants belonging to the genera *Azotobacter* (Sahoo et al., 2014), *Azospirillum* (Omar et al., 2009), *Rhizobium*, *Pantoea*, *Bacillus, Pseudomonas* (Sorty et al., 2016), *Enterobacter* (Nadeem et al., 2007), *Bradyrhizobium* (Panlada et al., 2013), *Methylobacterium* (Meena et al., 2012), *Burkholderia* (Oliveira et al., 2009), *Trichoderma* (Ahmad P. et al., 2015) and *cyanobacteria* (Joshi et al., 2020) have been reported to contribute in growth promotion of several crop plants.

Plant microbiome offers an abiotic stress protection mechanism to the host as the metabolic potential of microbiome is immense and it supplements the metabolic capacity of the plants to acquire nutrition and develop tolerance against stress. The phyto-microbiome is dynamic and its organization is sculpted by the degree and duration of the abiotic stress. The plant and its associated microbiome synergistically respond to abiotic stress for mutual survival and growth (Javaid et al., 2022). Under unfavorable environmental conditions, soil-dwelling microorganisms from the genera *Achromobacter*, *Azospirillum*, *Variovorax*, *Bacillus*, *Enterobacter*, *Azotobacter*, *Aeromonas*, *Klebsiella*, and *Pseudomonas* have been demonstrated to promote plant growth (Dardanelli et al., 2008; Belimov et al., 2009; Ortiz et al., 2015; Kaushal and Wani, 2016; Sorty et al., 2016). *Burkholderia phytofirmans* strain *PsJN* has been found to reduce salt stress in *Arabidopsis* (Pinedo et al., 2015) and maize (Naveed et al., 2014b), as well as drought stress in wheat (Naveed et al., 2014a).

The response of the phyto-microbiome to the abiotic stress largely influences the growth, tolerance, adaptation, and evolution of the host plant and microbes both. There is now mounting evidence that plant-associated microbes may prove to be instrumental in the sustenance of agriculture in times of drastic impacts of climate change. The phyto-microbiome architecture could be better utilized for abiotic stress alleviation of plants and the development of stress-tolerant crop plants if the ecological relationships of the plant-associated microbial diversity and mechanisms of their interactions are deeply understood. The potential options for overcoming crop plants’ productivity constraints in stress-prone environments include the selection, screening, and application of stress-tolerant microorganisms. The application of beneficial microorganisms as bioinoculants can be a good alternative for promoting plant growth under various types of abiotic stresses.

In this review paper, the ecology of phyto-microbiome is summarized, focusing on the beneficial microbes and their role during abiotic stress conditions. The physiological and molecular responses of phyto-microbiome against major stressors drought, heat, salinity and heavy metal toxicity are discussed to determine the role of the microbiome in the stress alleviation of plants. Ascertaining the potential of microbes in the development of stress-resistant plants, the paper further emphasizes modern strategies like introducing novel bio-inoculants, application of multi-omics technologies for gene modification, and phyto-microbiome-based genetic engineering as sustainable solutions to enhance agricultural productivity.

## 2. Ecological structure and function of phyto-microbiome

**Figure 1:**
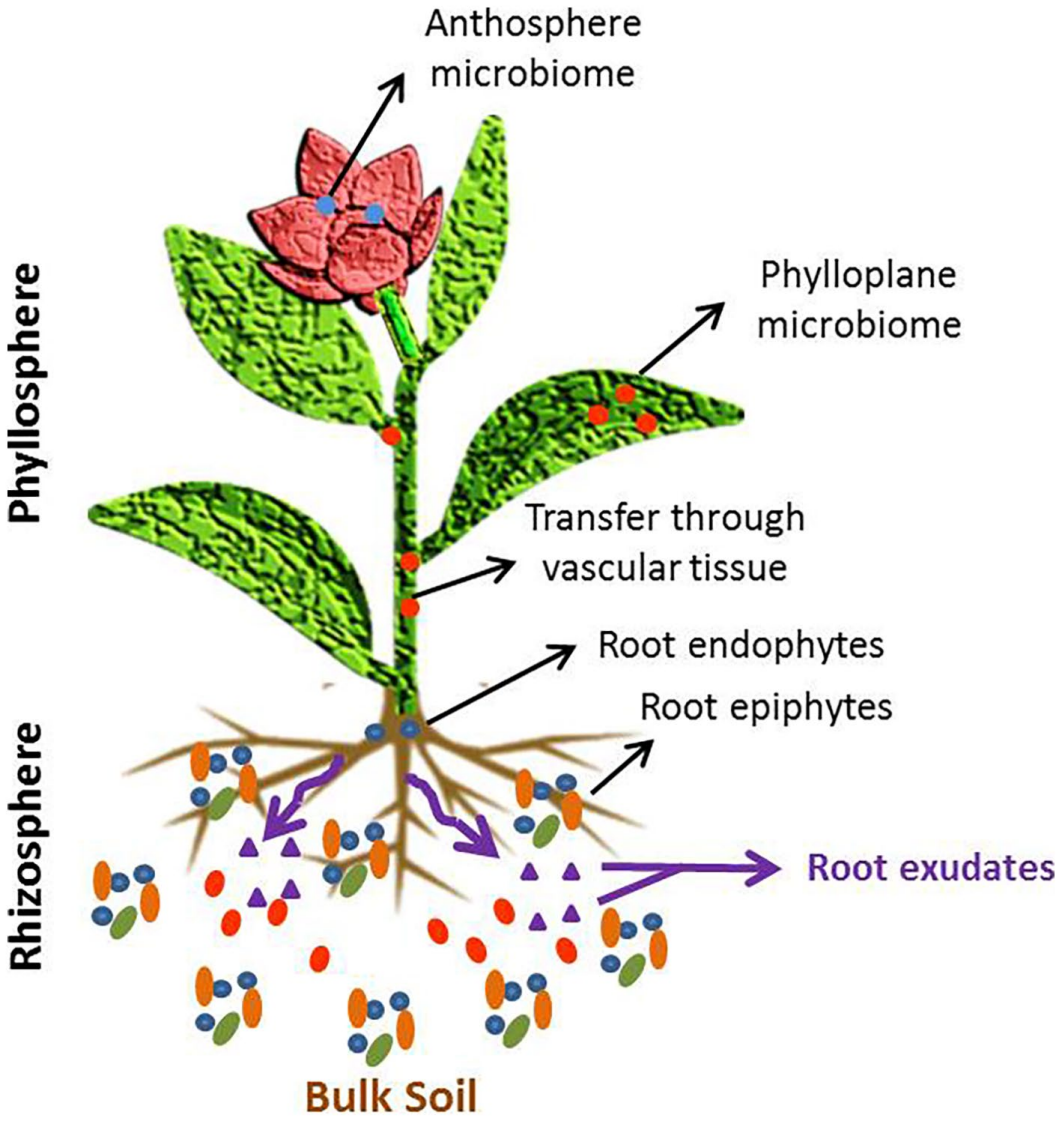
Structure of phyto-microbiome.

The phyto-microbiome is composed of taxonomically diverse communities including bacteria, archaebacteria, fungi, and viruses, which establish various ecological relationships with the host plant such as symbiosis, mutualism, and parasitism (Chialva et al., 2022). Microbiomes associated with plants are either epiphytic or endophytic, and colonize both niches–the phylloplane (above ground part) and rhizoplane (below ground part) (Santos and Olivares, 2021). Various plant compartments in which the microbes form their niche and colonize are depicted in Figure 1. Rhizospheric microbiome is selectively attracted and recruited by the host plant though rhizodeposition in which root exudates containing compounds like amino acids, carbohydrates, organic acids, fatty acids, siderophores and flavonoids, are secreted. These root exudates act as signals to establish communication between plant and specific microbes. The type of root exudates varies with plant’s genotype, innate immunity, signaling pathways and response to environmental conditions. The root exudate composition is instrumental in shaping the structure of phyto-microbiome assembly in the rhizosphere. The epiphytic interactions in the rhizoplane and phylloplane provide oppuortunity to the microbes to enter the tissues, systematically spread through vascular system and colonize other compartments as endophytes. The endophytic community structure is mostly substrate-driven and depends upon the allocation of resources in different plant compartments. Besides plant-microbe interactions, the microbe-soil interactions and microbe-microbe interactions significantly affect the plant growth (Santoyo, 2022).

### 2.1. Rhizobacteria

The soil microenvironment of the root region is rich in microbes because it contains a wide range of nutrients, minerals, and metabolites. The microbial colonization of the rhizosphere is significantly influenced by root exudates or substances that a plant root secretes (Kong and Six, 2012). Some microorganisms of the rhizosphere that aid in reducing abiotic stress include plant-growth-promoting rhizobacteria (PGPRs), act as beneficial microorganisms that adopt several strategies to reduce abiotic stress, including the production of phytohormones, a decrease in ethylene oxide levels, an increase in the dehydration response, and the induction of genes encoding antioxidant enzymes (Yang et al., 2009). Further, these bacteria contribute to the production of plant growth regulators like indole-3-acetic acid (IAA), deaminase, and 1-aminocyclopropane-1-carboxylic acid (ACC) that aid in enhancing plant growth (Glick, 2014). It was observed that the genera *Diazotrophicus*, *Bacillus*, *Pseudomonas*, *Azotobacter*, *Azospirillium*, *Rhizobium*, *Burkholderia*, *Gluconacetobacter* and *Serratia* are the most important rhizospheric inhabitants that help plants mitigate a variety of abiotic stresses (Backer et al., 2018). In order to reduce stress in rice, *Trichoderma harzianum* was used to increase aquaporin, dehydrin, and malondialdehyde (Pandey et al., 2016). Additionally, *T. harzianum* was used to increase the oil yield from salinity affected Indian mustard (*Brassica juncea*) which enhanced antioxidant synthesis, decreased Na + uptake, and improved nutrient uptake in plants (Cho et al., 2008). Rhizobacteria-induced drought endurance and resilience (RIDER) is defined as changes in the levels of phytohormones, defense-related proteins, enzymes, antioxidants, and epoxy polysaccharides on exposure to various stresses (Kaushal and Wani, 2016). These changes increase the plants’ resistance to abiotic stress (Raymond et al., 2004). IAA and ACC-deaminase production in barley and oats appeared to be improved using *Pseudomonas* sp. and *Acinetobacter* sp. (Lu et al., 2013). *Pseudomonas* sp. improved its ability to inhabit roots sideways is due to its capacity to produce exo-polysaccharides (EPS) stimulus, and increased salinity resistance in rice during germination (Rojas-Tapias et al., 2012). Actinomycetes are known to promote plant growth and lessen the damage caused under abiotic stress. They are able to grow under harsh environment such as high salinity, drought and high temperature (Grover et al., 2016). In the rhizosphere the Actinomycetes utilizes the nutrient and water more efficiently in the stressed soil as they possess the ability to cleave the rhizospheric soil particles and hence form strong bonds with the plants (Sandrini et al., 2022). These bacteria follow several mechanisms such as changes in root and cell wall morphology, 1-aminocyclopropane-1-carboxylic acid (ACC) deaminase activity, possess the ability to avoid oxidative damage, phytohormonal alterations, compatible solute production (glycine-betaine and proline) that promotes osmoregulation (Chukwuneme et al., 2020).

### 2.2. Phyllosphere bacteria

Phyllosphere is an ideal environment for microbes that harbors a huge variety of beneficial microbes belonging to bacteria, fungi and viruses. The performance of the plant is significantly influenced by the phyllospheric microbiome. These microbes also assist plants in purging contaminants. Additionally, they support the preservation of plant health and control the spread of plant pathogens. The long-distance transport process has a significant impact on the microbiota of plant parts that are distant from the soil or in other aerial parts of plants (Arun et al., 2020). When rice plants were stressed by drought, inoculating the plants with the plant growth-promoting, drought-tolerant *Bacillus altitudinis FD48* increased relative water content, chlorophyll stability index, and membrane stability index compared to control (uninoculated plants) (Awasthy et al., 2017).

### 2.3. Fungi

Many fungi inhabiting the rhizospheric soil have remarkable potential of degradation of various pollutants, thereby protecting the plants from abiotic stress (Shourie and Vijayalakshmi, 2022). Some fungi like Arbuscular mycorrhizal fungi (AMF) are obligate mycorrhizal fungi that form symbiotic relationships with vascular plants including halophytes. AMFs can sporulate in the rhizosphere as well as form vesicles and hyphae in roots. Plant growth is increased because of the excellent access to the soil surface area provided by the hyphal network that AMFs create. Through the effective translocation of nutrients, AMFs contribute to an improvement in plant nutrition. Additionally, they aid in enhancing the health of plants and the soil (Compant et al., 2010). Plant productivity is typically reduced by drought stress, in which AMFs assist plants in retaining growth and increasing yield. AMFs aid in increasing water uptake as part of the drought mitigation mechanism and aid the plant in enhancing nutrient uptake, which enables plants to withstand stresses (Jiang et al., 2016). It was found that the plant biomass, fruit yield, and shoot content of P, K, Cu, Fe, and Zn increased when a tomato plant was inoculated with *Funneliformis mosseae* under saline conditions (Chandrasekaran et al., 2021). Wheat plants inoculated with AMFs performed well under salt stress and the oxidative damage to the plants is significantly reduced (Hayat et al., 2010). Extremely low and high temperatures, however, were reported to inhibit the development of the extra radical hyphal network and AMF fungal activity, and decrease AMF fungal growth (Mathur and Jajoo, 2020). AMF helps plants grow their root system for water absorption at high temperatures to ensure high photosynthetic capacity and prevent damage to the photosynthetic apparatus. The inoculation of barley (*Hordeum vulgare* L.) with AMF led to improved growth, photosynthesis, osmotic homeostasis, and potassium uptake under low-temperature conditions, and *Glomus versiforme* was frequently more successful than *Rhizophagus irregularis* at boosting survival rates (Hajiboland et al., 2019). Vesicular Arbuscular Mycorrhiza (VAM) also alters the physiological, functional, and biochemical makeup of plants in ways that increase their ability to withstand various abiotic stresses. AMF inoculation in vegetables has been shown to boost biomass production and increase yield (Haghighi et al., 2015; Duc et al., 2018). The uptake of greater amounts of nutrients, leaf water potential, and stomatal conductance are all significantly influenced by AMF inoculation (Khan et al., 2013). When lettuce plants were inoculated with AMF, their abscisic acid (ABA) levels decreased, indicating that they were less stressed than uninoculated plants. Therefore, AMF inoculation modified the plant’s hormonal profile and physiology to make it more suited to saline conditions (Aroca et al., 2013).

### 2.4. Endophytes

Endophytes have symbiotic relationships with plants and live inside them for the entirety of their life cycles. Endophytes typically invade the seeds, roots, leaves, and stems of host plant, establish colonies in plant tissues and promote plant growth by enhancing nitrogen fixation, phytohormone secretion, and nutrient uptake. During periods of abiotic stress, endophytic microbes stimulate plant growth by various mechanisms such as osmolyte accumulation, induced systemic tolerance, production of phytohormones such as ABA, gibberellic acid (GA), cytokinins and IAA, ACC deaminase production for lowering ethylene. The endophytic *Arthrobacter* strains *EZB4*, *EZB18*, and *EZB20* inoculation increased the proline content in *Capsicum annum* L. exposed to abiotic stress (Sziderics et al., 2007). Salinity stress was alleviated by *Bacillus firmus* and *Bacillus* sp. in peanut, *Curtobacterium* sp. in soybean and rice, *Enterobacter ludwigii*, *Bacillus cereus* and *Micrococcus yunnanensis* in rice (Khan et al., 2019, 2020; Pal et al., 2021). The root fungal endophyte *Piriformospora indica* induced drought tolerance in Chinese cabbage (Sun et al., 2010) and salt tolerance in barley (Baltruschat et al., 2008), by boosting the levels of antioxidants.

## 3. Physiological and molecular response of phyto-microbiome against abiotic stress

The responses of crop plants against abiotic stresses are manifested as altered phenotypes at morphological, physiological, and biochemical levels. Plants have developed complex signaling mechanisms to counteract stress conditions and enable survival. The plant microbiome further supplements the metabolic capacity of the plants to combat stress conditions. Here, the major stress factors (drought, heat, salinity and heavy metal) are discussed for their impact, plant response, and the role of the microbiome in combating stress.

### 3.1. Drought stress

Severe drought stress leads to wilting, yellowing, discoloration, and leaf burning in plants. Plants have the inherent capability to respond to drought stress and they try to control the damage by complex mechanisms. They respond and regulate the drought stress by closing stomata, decreasing the surface area of succulent leaves, and increasing the roots. However, prolonged drought stress is known to stunt plant growth with a reduction in leaf size, and stems, production of a greater number of roots, decrease in RubisCO activity and photosynthetic pigments, reduction in seedling vigor, and decrease in seed germination. It reduces membrane potential and increases the concentration of reactive oxygen species (ROS) causing free radical damage and disruption of ATP synthesis (Shaffique et al., 2022).

#### 3.1.1. Drought stress response and signaling

Water scarcity is detected by the leaves and roots. However, the signals are transmitted majorly from roots to shoots. Plants sense and transmit water deficit through signaling activated by osmotic pressure, ROS and mechanical stresses, involving numerous sensing molecules. ABA is an important phytohormone that is produced in response to drought stress and plays a crucial role in adaptation to drought stress. ABA is mostly produced in vascular tissues and is transported via a transporter to various tissues. It induces stomatal closure and activation of stress-related genes that increase drought resilience (Kuromori et al., 2022). Water deficit induces the expression of enzymes of ABA biosynthetic pathway such as ZEP/ABA1, AAO3, cis-epoxy carotenoid dioxygenase (NCED3), and molybdenum cofactor sulfurase (MCSU/LOS5/ABA3). The binding of ABA with ABA receptor proteins PYR/ PYL/ RCAR initiates the ABA-dependent stomatal regulation pathway leading to the activation of Protein Phosphatases 2C (PP2C) and SNF1-Related Protein Kinases 2 (SnRK2). The transcription factors such as ABF, MYC MYB, NAC, ERF, bZIP, and DREB/CBF are activated, and they bind to nuclear targets resulting in the expression of drought stress proteins (Ali S. et al., 2022; Aslam et al., 2022).

Drought stress causes a variety of biochemical changes inside the host, including an excessive build-up of reactive oxygen species (ROS), which can harm different tissues and cellular components like nucleic acids and other biomolecules, leading to programmed cell death (PCD) (Hasanuzzaman et al., 2020). In addition to altering biogeochemical cycles like the nitrogen and carbon cycles and slowing down the breakdown of organic matter, drought stress can also cause a considerable decrease in plant absorption and translocation of macronutrients (K, N, and P). Drought stress also reduces the absorption of cations (Ca^2+^, K^+^, and Mg^2+^) leading to the inhibition of several vital enzymes (Farooq et al., 2009).

#### 3.1.2. Role of phyto-microbiome in drought stress

It has been discovered that plant-associated microbiomes secrete a variety of chemicals during drought, including phytohormones, osmolytes, and antioxidants, which increase plant drought tolerance. Apart from facilitating plant growth, phytohormones such as IAA, cytokinin, and gibberellins, can assist plants in coping with abiotic stresses. Interestingly, the primary mechanism of mitigation of drought stress by plant microbiome is by inducing drought stress-responsive genes and regulating phytohormones (Iqbal et al., 2022).

### 3.2. Heat stress

Heat stress leads to a decrease in cell water content, cell size, plant size, growth, and biomass. Severe heat stress leads to scorching and discoloration of leaves, fruits, and other plant parts. Heat stress also leads to the alteration of biomolecular composition. It has been seen that heat stress increases the concentration of amino acids while decreasing the concentration of starch, sugars, and lipids. Maltose concentration has been seen to be elevated (Dastogeer et al., 2022). At the molecular level, heat stress leads to protein denaturation and misfolding. The cell membrane fluidity is increased while membrane integrity is compromised (Sehar et al., 2022). The indirect effects are complex to decipher. The increase in temperature can cause previously unknown infections due to the growth of microbial pathogens and the emergence of newer more pathogenic strains (Velásquez et al., 2018). Continuous thermal stress can increase the deposition of reactive oxygen species (ROS) resulting in membrane depolarization and initiation of programmed cell death (Katano et al., 2018).

#### 3.2.1. Plant defense against heat stress

Plants possess inherent thermal tolerance known as basal heat tolerance while thermotolerance can also be acquired. Plants resort to short-lived or long-term adaptation strategies to combat heat stress. Some plants have leaf and bud shedding, annual flowering, or regenerative stage completion in winter as an adaptation to high-temperature habitats. Heat stress leads to the induction or activation of ion transporters, antioxidants, phytohormones and signal transduction elements. Late embryogenesis abundant (LEA) proteins are produced to protect against heat stress. To prevent acute heat injury and mortality, plants contain molecular stress memory states known as short-term acquired tolerance (SAT) and long-term acquired tolerance (LAT). Particularly, C4 and CAM plants adopt a variety of modifications to boost the process of photosynthesis - under heat stress. Intensive transpiration from leaves can prevent damage by lowering the temperature of the leaves by several degrees (Hasanuzzaman et al., 2013).

Heat stress leads to the production of reactive oxygen species and in response the production of antioxidants such as peroxidase (POX), ascorbate peroxidase (APX), glutathione reductase (GR), superoxide dismutase (SOD), and catalase (CAT) is triggered in plants. The superoxide anion radical is changed by SOD into H_2_O_2_ and O_2_, which are subsequently changed into water and oxygen by CAT and APX. In plant cells, GR plays a role in the regeneration of the reduced glutathione which is an essential antioxidant. These detoxification systems maintain cellular homeostasis and promote plant growth and development during heat stress (Zandi and Schnug, 2022). Plants activate a complex signaling system involving heat shock factors (HSFs) that control the transcription of heat shock genes, including the production of heat shock proteins (HSPs). HSPs help protect the plant by promoting the proper folding of proteins, preventing protein denaturation and aggregation, and facilitating the breakdown of defective proteins. Different types of HSPs are produced, such as Hsp60, Hsp70, Hsp90, Hsp100, and sHSPs, each has specific functions in maintaining cellular homeostasis and promoting thermotolerance. Overall, the synthesis and overexpression of HSFs and HSPs play a critical role in enabling plants to cope with high-temperature stress (Ul Haq et al., 2019).

#### 3.2.2. Induction of thermotolerance

**A** brief pre-exposure to mild heat stress, also called priming, might cause plants to develop thermotolerance. This brief exposure builds a molecular stress memory which allows quicker and higher expression of heat stress transcription factors (HSFs) that control the production of heat shock proteins (HSPs) and antioxidant genes (Khan et al., 2022). Under heat stress, HSPs work as molecular chaperones to preserve the structure and function of proteins (Jacob et al., 2017). As a result, stress memory enables primed plants to respond swiftly to heat stress and recover from the adverse effects of heat. Four isomers of HSFA1A, B, D, and E, are known as master regulators of heat stress. They trigger the expression of HSFA2. A group of heat stress response genes, known as memory genes are in turn amplified by HSFA2 (Friedrich et al., 2021).

#### 3.2.3. Phyto-microbiome in combating heat stress.

Plant growth-promoting microorganisms (PGPM) can induce thermotolerance in plants by the production of heat shock proteins and induction of structural changes in plants. Moreover, phytohormone production, nutrient mobilization, and nitrogen fixation are brought about by the PGPM. Rhizospheric microorganisms produce and secrete phytohormones like IAA, gibberellins, and cytokinins. Endophytic microorganisms modulate the levels of abscisic acid, salicylic acid, and jasmonic acid under multiple stresses. Auxins are required for cell division and differentiation, growth of root and shoot, and seed germination, gibberellins regulate embryogenesis, stem growth, flowering, and fruit ripening, and abscisic acid regulates cell division and fruit ripening. Cytokines are involved in seed germination, root and shoot development, while ethylene is involved in abscission, senescence, and reproductive development. PGPM which produce gibberellins stimulate plant growth and stress tolerance (Hakim et al., 2021). Plant-associated microorganisms known to secrete exopolysaccharide form a biofilm over the plant roots and make a protective barrier and facilitate nutrient supply. Exopolysaccharide-producing *Bacillus cereus* was found to increase root and shoot length, chlorophyll content, water-intake, flowering, and fruiting in tomatoes (Mukhtar T. et al., 2020).

### 3.3. Salinity stress

Salinity reduces nutrient and microbial diversity, organic matter, nitrogen, dissolved organic carbon, and microbial carbon biomass in soil. Additionally, it causes osmotic stress, disturbs the nutrient balance, reduces chlorophyll content, leaf area, and photosynthetic efficiency, and negatively impacts intracellular K^+^ influx. Salinity stress also affects several cellular enzymes involved in nitrogen metabolism and synthesis of amino acids and indirectly induces the accumulation of ROS, which could damage the plant cells.

#### 3.3.1. Salinity stress response and signaling

Salinity stress is perceived by cell surface receptors relaying the signals through secondary messengers like inositol phosphates and ROS, and the activation of proteins like calcium-dependent protein kinase (CDPK) and mitogen-activated protein kinase (MAPK) that regulate the expression and function of numerous genes. Transcription factors play a pivotal role in imparting resilience towards salinity stress through modulation of expression of the salinity stress genes (Hasanuzzaman and Fujita, 2022).

#### 3.3.2. Role of phyto-microbiome in salinity stress

Plant microbiome employs several strategies to survive salinity stress including production of osmolytes, synthesis of extracellular proteases, and activation of Na+/H+ antiporter. They induce the production of plant growth hormones auxins, cytokinins, and gibberellins. Under salinity stress, the hormone ABA production is stimulated which reduces salinity stress by promoting osmolyte build-up in root vacuoles and the uptake of Ca^2+^ and K^+^ (Chen et al., 2022). Cytokinins maintain plant totipotent cells in the shoot and root apical meristems. Under abiotic stress, ethylene is known to accumulate in plants. Ethylene is an essential hormone and signaling molecule which plays key role in growth, seed germination and ripening, root hair elongation, and leaf senescence. However, high ethylene concentration has a detrimental effect on plants. Plant growth promoting bacteria (PGPB) produce ACC deaminase which lowers ethylene by converting ethylene precursor to ammonia and ketobutyrate. In response to abiotic stress, microorganisms develop biofilms, which cover the roots and keep them from drying out. They also foster optimal microenvironments for interactions between plants and microbes (Hakim et al., 2021).

Highly soluble organic substances such as sugars, sugar alcohols, glucosyl glycerol, betaines, amino acids, and tetrahydropyrimidine are produced or accumulated by bacteria. These osmolytes help in maintaining the osmotic pressure of the cells under salinity. At the same time, plant cells also assimilate osmolytes such as disaccharides, oligosaccharides, sugar, alcohols, glycine, betaine, proline, and glutamate which in turn help in the survival of plant microbiome during salinity stress (Kumar et al., 2020).

Salinity stress impacts the uptake of Nitrogen (N), Phosphorus (P), Potassium (K), and water, leading to huge reduction in crop yields. PGPB improve nitrogen uptake and bioavailability of phosphorus by acidification and chelation. Similarly, the bioavailability of microelements such as Cu, Fe, Mn, Zn is also increased. Potassium-solubilizing bacteria such as *Burkholderia* convert potassium into a bioavailable form. Salinity reduces iron availability and exacerbates iron deficiency in plants. Iron is essential for the activity of several plant enzymes and for the synthesis of chlorophyll (Teo et al., 2022). Siderophore-producing PGPB contribute significantly to Fe accumulation in roots and to its transportation to leaves (Yasmin et al., 2020; Sultana et al., 2021). Endophytic *Streptomycetes* that produce siderophores have been shown to increase root and shoot biomass as a result of improved Fe supply. Siderophore-producing PGPB have been demonstrated to increase salt tolerance (Afzal et al., 2019; Saeed et al., 2021).

*Trichoderma harzianum* reduced salt stress in plants by upregulating monodehydroascorbate reductase generating ACC-deaminase, as supported by mutant experiments (Brotman et al., 2013). In salty soil, *Pseudomonas* sp. and *Acinetobacter* sp. were found to increase IAA and ACC-deaminase synthesis in barley and oats (Chang et al., 2014). *Streptomyces* sp. strain *PGPA39* was found to reduce salt stress and promote development in ‘Micro-Tom’ tomato plants (Palaniyandi et al., 2014). Tolerance in rice was increased against salt stress by inoculation of *Pseudomonas* sp. (Sen and Chandrasekhar, 2014) and against salt and high boron stress by inoculation of *Bacillus pumilus* (Khan et al., 2016).

### 3.4. Heavy metal stress in plants

Heavy metals pollutants such as Mercury (Hg), Arsenic (As), Cobalt (Co), Manganese (Mn), Iron (Fe), Cadmium (Cd), Nickel (Ni), Zinc (Zn), Copper (Cu), Chromium (Cr) and Lead (Pb) are released into the environment thorugh anthropogenic activities like growing industrialization, intensive agriculture, and urbanization (Ayangbenro and Babalola, 2017; Kurniawan et al., 2022). The uptake of an excessive amount of heavy metals by crop plants from the contaminated soil affects plant health due to toxicity and considerably reduces the yield.

Heavy metals impact the growth and physiological processes either directly by inhibiting cytoplasmic enzyme activity and inducing oxidative stress, or indirectly by altering the phyto-microbiome structure and functions (Dotaniya and Saha, 2016).

#### 3.4.1. Heavy metal stress response and signaling

Plants have evolved various strategies to detect and respond to heavy metal stress in their environment through complex stress signaling processes that involve multiple pathways and mechanisms. The uptake and transportation of heavy metals in plants depend upon several transporters and proteins which help in their sequestration, intracellular or tissue compatrmentalization and detoxification. ATP-driven pumps HMA (Heavy Metal ATPases) are found on the plasma membrane and tonoplast (vacuolar membrane). They transport heavy metal ions such as such as Cu, Zn Cd and Pb across membranes and facilitate the sequestration of heavy metals into vacuoles or their extrusion from the cytoplasm. ZIP transporters (Zrt/Irt-like Protein) are involved in the uptake of essential metals such as Zn, Fe, Mn and Cu, but they can also transport toxic metals like Cd and Pb on exposure. ZIP transporters are located in the plasma membrane and are responsible for the uptake of these metals from the soil into the root cells. NRAMP transporters (Natural Resistance-Associated Macrophage Protein) are involved in the uptake and translocation of many divalent metal ions such as Fe, Mn, Zn and Cd. They are found in the plasma membrane and endomembranes of plant cells. NRAMP transporters have been shown to play a role in metal distribution within the plant and in metal detoxification processes. ABC transporters (ATP-Binding Cassette) constitute a large family of proteins involved in various cellular processes, including heavy metal transport. Some ABC transporters are known to transport heavy metals such as Fe, Cu and Zn. They are also present in the plasma membrane and other intracellular membranes.

It has been seen that initial abiotic stress signaling pathways are shared among the different types of abiotic stress. The heavy metal stress signaling involves production of Reactive Oxygen Species (ROS) such as superoxide radicals (O^2−^) and hydrogen peroxide (H_2_O_2_), in response to heavy metal induced cellular damage in plants. They act as secondary messengers in the signaling pathways. Mitogen-Activated Protein Kinase (MAPK) signaling pathway is one of the major pathways activated by heavy metal stress. MAPKs modulate the expression of stress-responsive genes, including those involved in metal detoxification and ROS scavenging. Phytochelatins (PCs) are small peptides synthesized in response to heavy metal stress by the enzyme phytochelatin synthase (PCS), which conjugates glutathione molecules to form PC complexes. PCs play a crucial role in heavy metal detoxification by chelating heavy metals and sequestering them into vacuoles, preventing their toxicity. Metallothioneins (MTs), low molecular weight, cysteine-rich proteins, are induced by heavy metal stress, which have a high affinity for heavy metals and can bind and sequester them, thereby reducing their toxicity. MTs are involved in metal homeostasis and play a protective role against heavy metal stress in plants. Heavy metal stress triggers changes in intracellular calcium (Ca^2+^) concentrations, leading to calcium signaling. Calcium ions act as secondary messengers and regulate Calcium-dependent protein kinases (CDPKs) are activated by increased calcium levels. CDPKs modulate the expression of stress-responsive genes. Heavy metal stress can also activate the abscisic acid (ABA) signaling pathway in plants, which promotes the expression of stress-responsive genes, thereby improving plant tolerance to heavy metals. Several transcription factors specifically AP2/ERF, MYB, WRKY, and NAC families are involved in regulating the expression of stress-responsive genes under heavy metal stress and regulate the expression of heavy metal response genes (Tiwari and Lata, 2018; Keyster et al., 2020).

#### 3.4.2. Role of phyto-microbiome in heavy metal stress

Phyto-microbiome plays a crucial role in heavy metal stress mitigation in plants. These microorganisms render the protection to plants from harmful effects of heavy metals through many ways, such as heavy metal sequestration or biosorption, nutrient mobilization and solubilization, heavy metal transformation and detoxification, induction of stress tolerance.

Microbes efficiently bind and sequester heavy metals, preventing their accumulation in plant tissues. They promote the immobilization and containment of heavy metals in the soil, reducing their bioavailability to plants. Microbial enzymes can mobilize and solubilize the essential nutrients in the soil, making them more accessible for plant uptake. They can convert insoluble compounds into soluble forms, increasing their bio- availability and reducing heavy metal toxicity. Microbes also transform and detoxify heavy metals through processes like reduction, oxidation, and methylation.

Plant-associated microorganisms induce systemic resistance and enhance the stress tolerance of plants. They can stimulate the production of plant growth-promoting hormones, antioxidants, and other protective compounds, which help plants to cope with heavy metal stress. It is important to note that the effectiveness of plant-associated microorganisms in heavy metal stress mitigation can vary depending on the specific microorganism, plant species, and environmental conditions. Syed et al. (2023) isolated strains of *Pseudomonas fluorescence* and *Trichoderma* spp. from heavy metal contaminated soil and improved the growth and yield of chickpea by lowering Cd uptake (; Oubohssaine et al., 2022; Syed et al., 2023) isolated rhizobacterial strains from heavy metal contaminated mining sites and studied their application on growth of *Sulla spinosissima* L. in a highly multi-polluted toxic soil. They observed that the strain LMR291 (*Pseudarthrobacter oxydans*), LMR340 (*Rhodococcus qingshengii*), LMR249 (*Pseudarthrobacter phenanthrenivorans*), and LMR283 (*Pseudomonas brassicacearum*) substantially improved all the growth parameters of *Sulla* plants, their photosynthetic pigments, and their antioxidative enzymatic activities (Oubohssaine et al., 2022).

## 4. Bioinoculants for alleviating abiotic stresses

Bioinoculants are formulations of microorganisms which can be inoculated in crop plants for facilitating growth and enhance production. These comprise of living or quiescent cells of specific microbial strains that benefit host plant by mechanisms such as facilitating nutrient acquisition, releasing plant growth hormones, and other biological activities like pest control. Additionally, bioinoculants may also be used to mitigate the harmful effects of abiotic stress (Benidire et al., 2020). Several theories have been proposed to elucidate the mechanisms of beneficial effects of bioinoculants including production of phytohormones, biofilm, EPS, and ACC deaminase (Tittabutr et al., 2013; Ansari et al., 2019; Goswami and Deka, 2020), production of antioxidants (Singh et al., 2019), cryoprotectants, heat shock proteins, solubilization of minerals such as phosphorus (P), potassium (K), and zinc (Zn), nitrogen (N) fixation, production of siderophores (Ferreira et al., 2019), antibiotics (Jin et al., 2021), hydrolytic enzymes such as proteases, cellulases, chitinases, and β-glucanases (Veliz et al., 2017), and volatile compounds (Harun-Or-Rashid and Chung, 2017). Some microbes also improve the induced systemic resistance (ISR) and systemic acquired resistance (SAR) and thereby help in alleviating multiple stresses in plants. Table 1 summarizes the effects of microbial inoculants on mitigation of abiotic stresses.

**Table 1:** Effects of various microbial inoculants in reducing abiotic stress and improving plant stress resistance.

Bioinoculants	Target plants	Effect on target plants	Effect on plant development	References
Salinity stress				
*Microbacterium oleivorans*, *Rhizobium massiliae*	*Capsicum annuum* L.	AA, ACC deaminase and siderophore production	Plant height, weight, and chlorophyll contents significantly enhanced	Hahm et al. (2017)
*Bacillus pumilus*	*Zea mays* L.	IAA, ACC deaminase activity, P-solubilization, EPS production and higher osmoprotectants and malondialdehyde production	Increased root and shoot dry weights	Mukhtar S. et al. (2020)
*Azotobacter salinestris*	*Sorghum bicolor* L.	increased ACC deaminase, salicylic acid, proline, and EPS production	Significant enhanced in growth parameters, chlorophyll, total carbohydrate, proline, and macro-elements content	Omer et al. (2016)
*Bacillus pumilus*	*Oryza sativa* L.	IAA, ACC deaminase, P-solubilization, proline aggregation, and EPS production	increased plant height, plant fresh, and dry weight, chlorophyll and carotenoids content	Ben Mahmoud et al. (2020)
Drought stress				
*Trichoderma* and *Pseudomonas*	*Oryza sativa* L.	the production of antioxidant enzymes such as peroxidase, glutathione peroxidase, ascorbate peroxidase, and glutathione	Promotes development of plants	Singh D. P. et al. (2020)
*Bacillus amyloliquefaciens* (MMR04)	*Pennisetum glaucum* L.	Reduced expressions of DREB-1E (drought–responsive) and ERF-1B (ethylene-responsive)	Promotes growth of the plants	Murali et al. (2021)
Temperature stress				
*Pseudomonas vancouverensis* and *Pseudomonas fredericksbergensis*	*Solanum lycopersicum* L.	Reduced ROS concentration, membrane damage and	Improved plant growth, and robustness in cold stress	Subramanian et al. (2015)
*Bacillus amyloliquefaciens* and *Brevibacillus laterosporus*	*Oryza sativa* L.	Increased proline, chlorophyll. Decreased leaf MDA content and electrolyte leakage	Increased overall plant growth in cold stress	Kakar et al. (2015)
*Lysinibacillus fusiformis YJ4* and *Lysinibacillus sphaericus YJ5*	*Zea mays* L.	Raising the amount of total phenolic content, osmolytes, antioxidant enzyme, and phytohormones	Enhanced growth of plants	Jha and Mohamed (2022)
Heavy metals stress				
*Enterobacter* sp.	*Pisum sativum* L.	IAA, siderophore production	Increased growth parameters, xanthophyll, carotenoid, and chlorophyll content	Naveed et al. (2020)
*Klebsiella* sp	*Zea mays* L.	IAA, EPS, catalase, phosphate solubilization	Increased shoot and root growth	Ahmad I. et al. (2015)

### 4.1. Bioinoculants for inducing salt stress tolerance in plants

In saline soils, plants experience two forms of stresses- nutrient stress and osmotic stress (Ashrafi et al., 2014). Salinity stress increases the production of ethylene hormone which is damaging and inhibits plant growth. Bioinoculants consist of microbes that produce ACC deaminase, which lowers ethylene concentration and maintain plant growth in saline conditions (Ansari et al., 2019). Numerous studies have shown that the ACC deaminase-producing microbes support plant growth in saline environments such as *Bacillus cereus* in *Vigna radiate* L. (mung bean) (Islam et al., 2016), *Bacillus pumilus* strain TUAT-1 in *Oryza sativa* L. (rice) (Win et al., 2022) and *Enterobacter* strain G in *Cajanus cajan* L. (Anand et al., 2021).

### 4.2. Bioinoculants for inducing drought stress tolerance in plants

Drought stress directly affects the water relations in plants and exters huge impacts on plant physiology. Bacterial IAA promotes the production of ACC deaminase, which dissociates one of the ethylene precursors and delays the onset of senescence in drought stressed plants (Uzma et al., 2022). Microbially produced ACC deaminase resists plant root drying by degrading ACC and reducing the level of ethylene in the plant cell (Ngumbi and Kloepper, 2016). Like auxin, cytokinin is another plant hormone that is important in preventing early leaf mortality during water shortages. Microbes also increase the synthesis of the endogenous stress hormone ABA, which is crucial for the plant’s drought resistance. Higher levels of endogenous ABA increase root-water conductivity by upregulating the expression of aquaporins (Goswami and Deka, 2020). Microbial inoculation also increases the synthesis of antioxidant enzymes in the plant, which helps the plant to enhance drought tolerance by decreasing ROS and increasing the production of antioxidant enzymes. Batool et al. (2020) showed that inoculating potatoes with *Bacillus subtilis* HAS31 reduced ROS production and mono-dehydroascorbate (MDA) production while increasing catalase, peroxidase, superoxide dismutase, and total soluble sugar in drought-stressed environments.

### 4.3. Bioinoculants for inducing temperature stress tolerance in plants

Heat stress has an impact on plants at several growth stages, including seed germination and reproduction, on a physical, physiological, and biochemical level. During high-temperature stress, seed germination rate and stand establishment are reduced due to the disturbed activity of enzymes involved in the breakdown of starch and synthesis of ABA & GA (Begcy et al., 2018). High temperatures have a significant impact on the photosynthetic process because they cause thylakoid disorganization, grana swelling and loss, a decrease in the activity of the electron acceptor and donor sites of photo-system (PS) II, and a decrease in the activity of enzymes such as RuBisCO (Hassan et al., 2020). Several microbes that are utilized as bioinoculants can withstand extremely high and extremely low temperatures. The temperature stress tolerance mechanisms include synthesis of heat and cold shock proteins, biofilm formation, and production of osmo-protective chemicals (Bruno et al., 2020). Inoculating *Solanum lycopersicum* L. (tomato) under chilling stress with *Trichoderma harzianum* was shown to increase photosynthesis and growth rate by lowering lipid peroxidation, electrolyte leakage, reducing ROS concentration, increasing leaf water and proline concentration (Ghorbanpour et al., 2018).

### 4.4. Bioinoculants for inducing heavy metal toxicity tolerance in plants

Heavy metals are absorbed by plants from contaminated soil through roots and are translocated to aerial parts through xylem, where they are bioaccumulated and impose considerable toxicity. Bioinoculants alleviate heavy metal toxicity by producing microbial siderophores for metal chelation, and phytohormones that boost the antioxidative enzymes in plants (Nazli et al., 2020). PGPB that are heavy metal tolerant (HMT) not only lessen the harmful effects of heavy metals but also encourage plant development in such conditions. Inoculation with the HMT-PGPB consortium increased the growth of *Sorghum bicolor* L. plants while also lowering the bioavailability of heavy metals Cu, Cd, Pb and Zn (El-Meihy et al., 2019). The inoculation of *Mucor* sp., *Klebsiella pneumoniae*, *Bacillus pumilus*, *Klebsiella* sp., and *Enterobacter* sp., also considerably reduced heavy metal contamination and improved plant growth (Ma et al., 2015; Karthik et al., 2016; Pramanik et al., 2017; Zahoor et al., 2017; Mitra et al., 2018).

## 5. Multi-omics approaches to mitigate abiotic stress in plants

Omics refers to the modern day technologies that give a deep insight into the metabolism, genomics and transcriptomics processes occurring in plants and hence the multi-omics approaches are advantageous in plant improvement against abiotic stresses. The understanding and sequencing of the whole plant genome in *Arabidopsis thaliana* L. have proved the potential benefits of omics tools. Various other plants such as rice, maize and soyabean possess a complicated genome, which have been fully sequenced by the use of omics technology (Ali A. et al., 2022). Numerous studies suggest that under abiotic stress not all genes are turned on or off at the same time, due to which the plant metabolism becomes complicated to understand and hence the phenotype cannot be determined by the genotype (Jha et al., 2019; Singh N. et al., 2020). Therefore, the amalgamation of proteomics, genomics transcriptomics, metabolomics, epigenomics, ionomics, interact omics, phenomics could help in identification of the candidate genes and improve the productivity of various crops under abiotic stress (Kumar et al., 2022).

The plant-microbe interactions are better understood due to the recent development in the various omics tools and sequencing technologies involving the regulation of gene expression and biodiversity (Sandrini et al., 2022). The characterization of beneficial microbes associated with plants and their functions along with the knowledge of rhizospheric science are possible due to the microbiome based multi-omics studies (White et al., 2017). The integrated omics approaches, computational and synthetic biology along with latest advances in high throughput culturing are providing significant knowledge about the structure and function of diverse natural microbiomes and providing avenues for artificially engineering the microbial communities and hence improving the crop growth, protection against pathogens and several abiotic stresses (Trivedi et al., 2021). Plants’ regulatory networks function to induce protective genes while inhibiting the negative regulators activated by abiotic stress factors. These regulations are responsible for the restoration of cellular homeostasis during the stress phase of plant cells. Multi-omics approaches help to integrate cellular processes at different levels based on systems biology knowledge (Katam et al., 2022).

### 5.1. Transcriptomics in abiotic stress

Transcriptome studies are a novel approach to understand the response of plants to abiotic stresses. Next-generation sequencing (NGS) and parallel RNA sequencing (RNA-Seq) are the two most promising techniques that open a new dimension of biological research to identify the networks among genes that actually respond to stress (Janiak et al., 2016). The advent of high-throughput omics techniques and rapid advancement in post-genomic epoch, specifically the next-generation sequencing (NGS), molecular characterization and modeling have proved beneficial in improving the efficiency and resilience of crop plants under abiotic stress (Pandey et al., 2021). The benefits of plant associated microbes and their communities are well understood by the use of NGS on DNA extracted from soil and rhizosphere and hence lead to better knowledge of their diversity, structure, abundance and important microbes (Alawiye and Babalola, 2019). Also the clustered regularly interspaced short palindromic repeats (CRISPR/Cas9) is an important technology and serves to knockout non-transgenic plant and microbe mutants, characterize symbiosis-related protein, plant traits that sustain beneficial microbiome, various genetic factors and identification of candidate genes responsible stress tolerance and further assigning them specific functions (Levy et al., 2018; Khatabi et al., 2019). The overexpression of transcription factors increases the expression of genes that encodes enzymes and chaperones associated with endoplasmic reticulum stress response, resulting in an increase in rate of photosynthesis and tolerance to drought-like abiotic stress (Wang et al., 2018).

Expressed sequence tags (ESTs), microarray, Affymetrix GeneChip technology, and serial analysis of gene expression (SAGE) have been used to elucidate the function of various genes associated with abiotic stress (Varshney et al., 2009; Deokar et al., 2011; Le et al., 2012). Chickpea genotype microarray study shows 210 differentially expressed genes (DEGs) and numerous differentially expressed unigenes under drought stress (Wang et al., 2012). Global transcriptome profiling of the root tissues of drought-stressed lentils, chickpeas, and ground nuts identified differentially expressed genes (DEGs) involved in different energy metabolism pathways like TCA cycle, glycolysis cycles mediated by transcription factors like WRKY, zinc finger family protein, bHLH, NAC, AP2/ERF and MYB protein domain family (Brasileiro et al., 2015; Singh et al., 2017). Genomics studies showed that ZmWRKY40 and ZmNF-YB2 genes encode a transcription factor that helps in resistance to drought in maize plants (Nelson et al., 2007; Zhang et al., 2008; Gangola and Ramadoss, 2020). In *Arabidospsis* bZIP was identified and it was associated with drought, salt, and cold tolerance by increasing oxidative enzyme level (Zong et al., 2018).

### 5.2. Meta-transcriptomics and metaproteomics

Meta-transcriptomics helps in the assessment of expressed genes (Nilsson et al., 2019). Studies related to ecology of microbial communities were possible by the sequencing of transcripts (RNA-seq) (Marcelino et al., 2019). However, interpretation of RNA-seq results is a tedious process but the advancement of databases and the increasing availability of annotated transcriptomes in curated databases as well as development of a robust *de novo* RNA-seq assembler can help in making the explanation of the result easier (Kuske et al., 2015). Metaproteomics provides functional data and suggests about the complex matrix such as specific soil sample. It emphasizes the study of proteins present in a biomass (Sandrini et al., 2022). It is an important phenomenon that helps in recognition of metabolic pathways, characterization of biological processes, plant-microbe interactions, their structure, function, significance, dynamics, and r egulation of symbiosis and molecular basis of cell communication (Khatabi et al., 2019).

### 5.3. MiRNA-omics in abiotic stress

Plant produces miRNAs (micro-RNAs) that are post-transcriptional gene-expression regulators, which help them to survive under stress conditions (Zhang and Wang, 2015). By using computational tools like screening of small RNAs library, different drought stress-responsive miRNAs were identified from *Arabidopsis*, rice, and sugarcane (Liu et al., 2008; Zhou et al., 2010; Gentile et al., 2015). Fourteen different stress-inducible miRNAs were identified from *Arabidopsis* and among them, miR168, miR171, and miR396 responded to all of the different types of stresses (Liu et al., 2008). In rice 18 cold-responsive miRNAs were identified by (Lv et al., 2010) most of which were downregulated by the cold stress (4°C) and it was supposed that miRNAs were ubiquitous regulators in rice. While on the other hand, due to cold stress sharp increase in the expression of *miR812q* in rice plants was observed in starting of the reproductive phase (Jeong et al., 2011). High-throughput sequencing revealed that 31 cold stress-induced genes were upregulated and 43 miRNAs were downregulated in tea (*Camellia sinensis* L.) (Zhang et al., 2014). In *Arabidopsis* roots enhanced expression of a few miRNAs like miR156g, miR157d, miR158a, miR159a, miR172a,b, miR391, and miR775 were observed, under low oxygen stress situations (Moldovan et al., 2010). In wheat *Tae-*miR6000, miR156, miR159, miR164, miR167a, miR171 and miR395 were identified as UV-B responsive microRNAs (Wang et al., 2013).

The functions of miRNAs in response to abiotic stresses as well as plants’ development are determined by using artificial miRNAs (amiRNAs), which could be useful to design the strategies for silencing endogenous genes and inhibit the expression of target genes (Zhang F. et al., 2022). Technologies such as Real-Time Quantitative Reverse Transcription Polymerase Chain Reaction (qRT-PCR) and microarrays suggested that abiotic stress conditions induce miRNA expression profiles and are diverse among plant species (Begum, 2022).

### 5.4. Metabolomics in abiotic stress

Metabolomics technology provides a chemical profile of thousands of compounds and involves the use of high-pressure liquid chromatography along with high resolution mass spectrometry (LC–MS), gas chromatography and mass spectroscopy (GC–MS) and nuclear magnetic resonance (NMR) spectroscopy for characterization of stress induced metabolites (Crandall et al., 2020). The suitability of the selection of the technique depends on the speed, sensitivity, and accuracy of the method used (Ghatak et al., 2018). Due to stress conditions plants can adapt to the stress or tolerate the stress. Mostly metabolomics studies focused on the comparative analysis of stress-susceptible and stress-tolerant responses of plants. Along with amino acids other metabolites like sugar, phenolic compounds, and organic acid also play an important role in plant abiotic stress (Dawid and Hille, 2018; Ghatak et al., 2018). The metabolomics and other -omics technologies, allowed a detailed and in-depth analysis of plant stress as the result of the alteration of metabolites and gene expressions (Anzano et al., 2022). It was observed that under stress conditions plants activate high proline production but under the stress recovery phase, it undergoes proline catabolism (Krasensky and Jonak, 2012). Metabolomics was used to study phytohormone response against salt stress as a targeted approach in roots and shoots in *Arabidopsis* seedlings (Šimura et al., 2018) and non-targeted approach in maize (Richter et al., 2015). Metabolomics revealed the contribution of overexpression of GmGSTU4, responsible for production of glutathione transferases (GSTs) and increase the glutathione biosynthesis under salt stress in transgenic tobacco (Kissoudis et al., 2015). In mycorrhizal roots the variations in the metabolomics profile have been observed leading to the identification of potential primed compounds that are involved in improved stress tolerance in mycorrhizal plants (Rivero et al., 2018; Bernardo et al., 2019). Metabolomics play an important role in studying the exudates released by the roots in the rhizosphere which serve as a significant feed source for the microbes that are associated with the roots as compared to the surrounding soil that is poor in nutrients (Escudero-Martinez and Bulgarelli, 2019). A major issue in metabolomics is that a huge variety of potential metabolites are present in any given sample and due to the limited extent of public metabolite reference databases assigning a measured metabolite to a specific organism or condition, it becomes difficult to correlate the metabolite production to a particular stress (Khatabi et al., 2019).

### 5.5. Genomics in abiotic stress

Abiotic stress studies on plants include many cellular processes like sensing, signaling, transcription, transcript processing, translation, and post-translational protein modifications. These studies ultimately boost crop productivity and agricultural sustainability through genetic, chemical, and microbial approaches (Zhang H. et al., 2022). The effect of different abiotic stress in plants was monitored quantitatively by using imaging technology along with the support of information technology. Various phenotypic expressions of plants are useful quantitative phenotypic tools, as genotypic changes lead to the expression of phenotypes. By using a combination of genomics and data science, we can analyze the plant stress responses under different combinations of environmental stress (Zandalinas and Mittler, 2022). In this method target sequences can be designed and introduced into the most appropriate vectors. DNA, RNA, or RNPs like genetic cargo is selected for further delivery by (i) modifying the targeted sequence, (ii) regenerating the edited calli, and (iii) producing the edited plants (Farooqi et al., 2022).

### 5.6. Metagenomics

Metagenomics identifies the genomic diversity and functions of microbial genes (Solden et al., 2016) and suggests about the relative abundance and taxonomic composition (Singer et al., 2016). The plant-microbe complexity can be studied through next-generation DNA sequencing methods like 454 pyrosequencing and second and third generation sequencing platforms such as PacBio RSII Sequel, GridION, Illumina MiSeq, NovaSeq, GeneStudio, Oxford Nanopore MinION, PrometION, Ion Torrent PGM (Nilsson et al., 2019). Two key methods such as shortgun metagenomics and metabarcoding are employed to identify the microbial communities and compare them on the basis of composition, richness, evenness and assembly (Sharma et al., 2020).

### 5.7. Phenomics-manipulating plant root-associated microbiomes

The plant root-associated microbiome influences numerous plant traits, primary and secondary metabolites that act as growth substrate for few microbes and have antagonistic effect on the others, they act as signals that regulate the plant microbe interactions. Although few rhizospheric microbial species act as symbionts or growth promoting rhizobacteria are beneficial for the plants and helps in enhancing the plant pathogen defense and nutrition, few microbes may be parasitic and commensal (Lareen et al., 2016; Pascale et al., 2020; Chen et al., 2021). Therefore the study and classification of the complex interaction of plant microbiome and soil rhizosphere is important for developing novel approaches towards crop resilence against pathogens and environmental stresses (Zenda et al., 2021).

## 6. Dynamic role of fauna in plant microbiome functions

Some of the useful fungi serve as plant parasitic nematode hunters such as *A. avenae* is effective against *Ditylenchus* (plant-parasitic nematode) propagation and is an active bio-controlling agent in many parasitic nematodes and pathogenic fungi (Haraguchi and Yoshiga, 2020). Members of Protista are abundantly present in the plant rhizosphere and regulates various mechanism such as nutrient recycling, and interactions in the food web, promotes productivity (Hünninghaus et al., 2017), and lowers the total bacterial biomass (Krome et al., 2010). It also suppresses plant pathogens and boosts immunity of plants against various pathogens. They represent diverse modes of nutrition, wide range of prey interactions, and chemical communication (Mahmud et al., 2021; Solanki et al., 2022). Due to their numerous interactions, the protists cause significant changes in the structure and function of the microbiome. Also regulates auxin and cytokinin levels and hence possesses a significant effect on plant microbiome linked to several hormonal fluctuations (Krome et al., 2010). Earthworms also possess a strong impact on the soil microbiome. It is present in the soil rhizosphere and significantly adds to about 80% of the biomass in the soil macrofauna (Yasmin and D’Souza, 2010). Depending on the earthworm type and the micro-habitat, its impact can be positive, negative, or neutral on the diversity and enrichment of the microbial population (Afridi et al., 2022b). The activities of microorganisms are activated due to the release of acutaneous mucus (glycoprotein) by the earthworm that enhances the interactions. Due to the significant interaction between the microorganisms and the earthworm, there is improved microbial activity in the soil, increased availability of nutrients and increased carbon turnover (Bedano et al., 2019).

## 7. Plant microbiome engineering to combat abiotic stress

**Figure 2:**
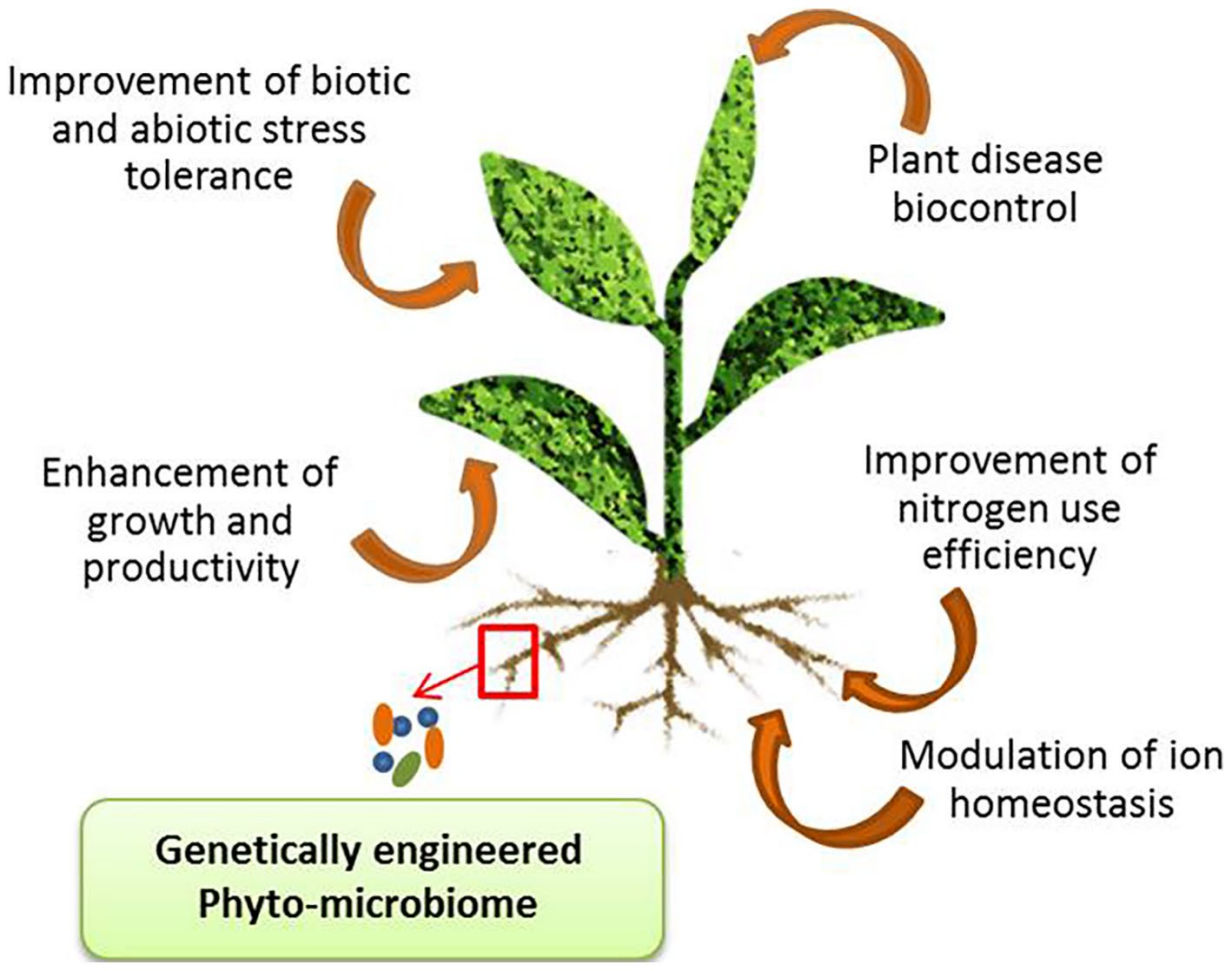
Major applications of plant microbiome engineering (PME).

Plant microbiome engineering (PME) is an important approach to promoting plant health, growth, and productivity under adverse environmental conditions (Afridi et al., 2022a). According to Parnell et al. (2016) soil microbiome is the next green revolution as it is serving as a promising tool that will meet the future global food demands. PME has been used to improve nitrogen use efficiency (NUE) in plants (Lau et al., 2022). Also, it is a beneficial technology in plants as it regulates the mechanism of hormones and specific antagonistic metabolite (rhizobitoxine) production that provides resistance against several pathogens, suppresses soil-borne diseases, and regulates nutrient availability in the rhizosphere (Rodríguez et al., 2020; Figure 2). Various approaches used for engineering the phyto-microbiome for developing abiotic stress tolerance in crop plants are summarized in (Table 2). The steps involved in engineering the plant microbiome include selection and engineering of the host-mediated multi-generation microbiome, inoculation of microbial communities as bioinoculants in the rhizosphere, soil, seedling/ seeds, mixed strain inoculation, tissue atomization, and direct injection in the plant tissues.

**Table 2:** Phyto-microbiome engineering approaches for abiotic stress tolerance in crop plants.

Plant growth promoting microbes	Host plant	Microbiome engineering approaches	References
*Brucella* sp. PS4	*Gossypium hirsutum* L.	Promotes pesticide degradation	Ahmad et al. (2022)
*B. subtilis* PM32	*Solanum tuberosum* L.	Biocontrol of fungal diseases	Mehmood et al. (2021)
*G. intraradices*	*Cucumis sativus* L.	Improves biomass, regulates salinity stress, enhances the production of antioxidant enzymes	Hashem et al. (2018)
*B. firmus* SW5	*Glycine max* L.	Regulates production of antioxidant enzymes, salinity tolerance	El-Esawi et al. (2018)
*B. subtilis* GB03	*Arabidopsis thaliana* L.	Monitors the import of sodium ions in root	Wang et al. (2016)
*Beauveria bassiana* and *Metarhizium brunneum* BIPESCO5	*Capsicum annuum* L.	Inhibit pathogenic *Fusarium* sp.	Jaber and Alananbeh (2018)
*A. pullulans* 490	*Solanum lycopersicum* L.	Biocontrol activity and helps in the production of biosurfactants	Köhl et al. (2020)
*B. pumilus* JPVS11	*Oryza sativa* L.	Regulates salt tolerance	Kumar and Sharma (2020)
Bacterial endophytes (*Bacillus* and *Brevibacillus*)	*Zea mays* L.	Enhances plant growth and development	Al Kahtani et al. (2020)
*Glomus mosseae*	*Triticum aestivum* L.	Controls osmotic potential and drought stress, regulates production of antioxidant enzymes	Rani et al. (2018)
*B. xiamenensis*	*Saccharum officinarum* L.	Improves the phytoremediation capacity	Zainab et al. (2021)
*P. geniculata*, *B. subtilis*, *B. siamensis*, *B. gelatini*, *B. ubonensis*, *B. territorii*	*Piper nigrum* L.	Antagonistic to soil-borne *Fusarium solani*	Lau et al. (2020)
*R. irregularis*	*Triticum aestivum* L.	Regulates heat stress, allocation of nutrient and nutrient composition in roots	Cabral et al. (2016)
*T. harzianum* Thar DOB-31	*Curcuma longa* L	Regulates production of Indole-3-acetic acid (IAA) and hydrogen cyanide	Vinayarani and Prakash (2018)
*Halomonas* sp.	*Avicennia marina* L.	Regulates heavy metal stress	Mukherjee et al. (2019)
Endophytic diazotrophic bacteria	*Sarracenia species* L.	Helps in nitrogen fixation	Sexton et al. (2019)
*B. safensis* SCAL1	*Solanum lycopersicum* L.	Regulates heat stress	Mukhtar et al. (2022)

### 7.1. Selection and engineering of the host-mediated multi-generation microbiome

The selection and engineering of the microbiome are important as it selects the microbial communities through the host and influences and modifies the traits of the host plant which further influences the microbiome (Mueller and Sachs, 2015). Hence there is a synergistic relationship between host-mediated microbial communities and contributes significantly to agricultural yield, biodiversity, and food security (Kaul et al., 2021). The “artificial selection of the ecosystem” was done to screen the plant biomass of *Arabidopsis thaliana* L.with the lowest (low selection lines) and highest (high selection lines) plant biomass which was earlier improved by the microbial community and their interactions with the plants for over 16 generations (Swenson et al., 2000). Different studies in *Arabidopsis thaliana* L. and *Brassica rapa* L. (Panke-Buisse et al., 2015) suggest a positive correlation between plant biomass and increase activity of microbial extracellular enzymes leading to soil nitrogen mineralization and suggesting the dynamic role of microbiomes to deal with numerous environmental and agronomic issues (Orozco-Mosqueda et al., 2018).

### 7.2. Inoculation of microbial communities as bioinoculants in the rhizosphere, soil, seedlings/seeds

Using microbial communities as bioinoculants finds wide applications such as plant growth, enhanced nutrient mobilization, stress resilience (Al Kahtani et al., 2020; Alok et al., 2020). The inoculation of different external strains from the rhizospheric soil can alter the structure of the microbiome. Different studies suggest their significant role. For example, the healthy oilseed crop was grown by using the bioinoculants on oil palm seedlings (*Elaeis guineensis* Jacq.), it modified the enzymatic and dynamic potential of rhizospheric microbes (Veeramachaneni and Ramachandrudu, 2020). Inoculation of *Agrobacterium* sp. *10C2* in *Phaseolus vulgaris* enhanced plant biomass and nodule formation by increasing antioxidant level, flavonoids, polyphenols, and phosphorus content in the beans, and also promoted colonization of beneficial rhizobacteria *Brevibacterium*, *Paenibacillus koreensis*, *Bacillus pumilus* and *Actinomyces* (Chihaoui et al., 2015). A group of biocompatible microbial communities enhanced the growth of maize seedlings under a greenhouse with low-phosphorus soil. They are the group of engineered bacteria that were studied for biofilm formation, phosphate solubilization, and root colonization (Magallon-Servin et al., 2020). In orchids, the inoculation of *Klebsiella oxytoca* and *Pseudomonas fluorescens* into *Dendrobium nobile* Lindl. promoted the vigor, growth, germination, and adaptability (Pavlova et al., 2017). Growth of tomato seedlings (*Lycopersicon esculentum* L. cv. Saladette) was significantly enhanced with the co-inoculation of two endophytic strains *Pseudomonas stutzeri E25* and *Stenotrophomonas maltophilia CR71* in the rhizosphere as compared to single inoculation (Rojas-Solís et al., 2018).

### 7.3. Mixed strain inoculation and tissue atomization

The significant effect of the microbiome depends upon its interaction, multiple mechanisms, and functions carried out by the microbial community. Mixed strain inoculation proved beneficial compared to single or no inoculation in Populus plants where additive incorporation of strains of *Pseudomonas* and *Burkholderia* isolated from *Populus deltoids* L. significantly improved the plant’s photosynthetic capacity and root biomass (Timm et al., 2016). Also, the response was analyzed by transcriptomics, and specific genes for *Pseudomonas* and *Burkholderia* were turned on through inoculation of each strain and mixture including genes that encode for stress (temperature & salinity) and regulate plant hormone (ethylene). The mixed inoculation was studied in various other genes involved in the synthesis of lipids, sulfate, and thiamine and also the comparison of mixed and single inoculation was studied on the metabolic profiling of the leaf (Timm et al., 2016). The tissue atomization technique proved successful in improving plant development through the bioengineering of plant microbiomes without any genetic manipulation. This technique was exploited by using an endophytic bacterium *Paraburkholderia phytofirmans PsJN* in the flowers of dicot and monocot plants and significant improvements were observed in the seed microbiome by vertical inheritance as well as growth parameters (Mitter et al., 2017).

### 7.4. Direct injection in the plant tissues

This technique helped in incorporating the antimicrobial properties in the plants susceptible to attack by pathogens. For example, Manuka (*Leptospermum scoparium* L.), a medicinal plant produces anti-microbial oil that is having a potential effect against pathogenic bacteria (*Pseudomonas syringae*; Orozco-Mosqueda et al., 2018). Bacteria were able to colonize and survive in the plant through direct inoculation of biocontrol agent and PGPB (*Arthrobacter agilis UMCV2*) which is given a direct injection in the stem of *Medicago trancatula* L. plant (Avilés-García et al., 2016). More efficient colonization will occur depending on the bioavailability of nutrients (Avilés-García et al., 2016). Certain plants for example *Zea mays* L., and teocinte have shown direct injection techniques using bacterial endophytes (Johnston-Monje and Raizada, 2011).

## 8. Conclusion

Microbial interactions with plants have multifaceted functions; on one hand, microbes help plants to maintain their growth and development by fixing, mobilizing, and producing nutrients, hormones, and organic phyto-stimulant compounds, while on the other hand, they induce local or systemic stress alleviation response mechanisms in plants to survive under abiotic stress conditions. Phyto-microbiome essentially helps crop plants in their adaptation and survival on exposure to abiotic stress via induced systemic tolerance. It plays a key role in determining the varying levels of phytohormones, defense-related proteins, enzymes, antioxidants, and secondary metabolites, which mediate the stress-signaling processes. Growth-promoting microbes use various mechanisms to enhance plant growth under stress conditions, which include the production of plant growth regulators, iron and zinc sequestration, phosphorus and potassium solubilization, siderophore production, atmospheric nitrogen fixation, secondary metabolite production, as well as facilitation of uptake of other essential macro- and micronutrients from the soil. Microbial communities exhibit excellent resilience towards environmental challenges. The microbiome also displays functional redundancy by which, in the wake of environmental stresses, one microbial taxon can be replaced by another that can survive the stress. In addition to enhancing the microbial community structure, the introduction of advantageous stress-tolerant microorganisms can help improve plant and soil health when exposed to abiotic stress.

Recent agricultural practices have provided evidence that microbial bio-inoculants such as PGPRs not only aid in reducing environmental stresses but also increase the production of a variety of crop plants including rice, maize, barley, and soybean. The bio-inoculants not only enhance crop yield by bolstering the plant’s defense mechanism and protecting it from abiotic stress such as drought, and salinity, but they also improve soil health. Recently, the use of a consortium of microorganisms in crop production has been promoted because a single bioinoculant might not be sufficient to protect plants from various stresses. The development of a novel and effective bio-inoculant formulation must be centered on the selection of effective native strains for improved outcomes and the bioinoculants must be tested at multiple sites prior to commercialization to avoid failure at field level.

Multi-omics tools and technologies have revolutionized crop improvement research for the development of abiotic stress-tolerant varieties. The potential of multi-omics approaches can be utilized to decipher the stress tolerance mechanisms governed by phyto-microbiome. Metagenomics and meta-transcriptomics offer huge potential to identify the complex microbial networks implicated in stress signaling and tolerance development. Genomics technologies enable high-throughput screening of beneficial microbes, leveraging gene modification and genetic engineering approaches for introducing abiotic stress resistance in plants. Plant microbiome engineering could be immensely beneficial in the development of strategies to improve plant health, enhance crop productivity, improve resistance against abiotic and biotic stresses, and achieve sustainable agriculture in an eco-friendly manner.

## Author contributions

AbS did the conception, design, editing, finalization, and submission of manuscript. AnS, SM, SC, PG, and AbS did literature survey, analysis and preparation of draft of the manuscript. AnS, SM, SC, and PG did revision and redrafting. All authors contributed to the article and approved the submitted version.

## Conflict of interest

The authors declare that the research was conducted in the absence of any commercial or financial relationships that could be construed as a potential conflict of interest.

## Publisher’s note

All claims expressed in this article are solely those of the authors and do not necessarily represent those of their affiliated organizations, or those of the publisher, the editors and the reviewers. Any product that may be evaluated in this article, or claim that may be made by its manufacturer, is not guaranteed or endorsed by the publisher.

## References

[ref1] Afridi M. S. Ali S. Salam A. César Terra W. Hafeez A. Sumaira A.. (2022b). Plant microbiome engineering: hopes or hypes. Biol. 11:1782. doi: 10.3390/biology11121782, PMID: 36552290 PMC9774975

[ref2] Afridi M. S. Javed M. A. Ali S. de Medeiros F. H. V. Ali B. Salam A.. (2022a). New opportunities in plant microbiome engineering for increasing agricultural sustainability under stressful conditions. Front. Plant Sci. 13:899464. doi: 10.3389/fpls.2022.899464, PMID: 36186071 PMC9524194

[ref3] Afzal I. Shinwari Z. K. Sikandar S. Shahzad S. (2019). Plant beneficial endophytic bacteria: mechanisms, diversity, host range and genetic determinants. Microbiol. Res. 221, 36–49. doi: 10.1016/j.micres.2019.02.001, PMID: 30825940

[ref4] Ahmad I. Akhtar M. Asghar H. Ghafoor U. Shahid M. (2015). Differential effects of plant growth-promoting Rhizobacteria on maize growth and cadmium uptake. J. Plant Growth Regul. 35, 303–315. doi: 10.1007/s00344-015-9534-5

[ref5] Ahmad S. Chaudhary H. J. Damalas C. A. (2022). Microbial detoxification of dimethoate through mediated hydrolysis by *Brucella* sp. PS4: molecular profiling and plant growth-promoting traits. Environ. Sci. Pollut. Res. 29, 2420–2431. doi: 10.1007/s11356-021-15806-1, PMID: 34374007

[ref6] Ahmad P. Hashem A. Abd-Allah E. F. Alqarawi A. A. John R. Egamberdieva D.. (2015). Role of Trichoderma harzianum in mitigating NaCl stress in Indian mustard (*Brassica juncea* L.) through antioxidative defense system. Front. Plant Sci. 6:868. doi: 10.3389/fpls.2015.00868, PMID: 26528324 PMC4604702

[ref7] Al Kahtani M. D. F. Fouda A. Attia K. A. Al-Otaibi F. Eid A. M. Ewais E. D.. (2020). Isolation and characterization of plant growth promoting endophytic bacteria from desert plants and their application as bioinoculants for sustainable agriculture. Agronomy 10:1325. doi: 10.3390/agronomy10091325

[ref8] Alawiye T. T. Babalola O. O. (2019). Bacterial diversity and community structure in typical plant rhizosphere. Diversity 11:179. doi: 10.3390/d11100179, PMID: 37186411

[ref9] Ali A. Altaf M. T. Nadeem M. A. Karaköy T. Shah A. N. Azeem H.. (2022). Recent advancement in OMICS approaches to enhance abiotic stress tolerance in legumes. Front. Plant Sci. 13:952759. doi: 10.3389/fpls.2022.952759, PMID: 36247536 PMC9554552

[ref10] Ali S. Tyagi A. Park S. Mir R. A. Mushtaq M. Bhat B.. (2022). Deciphering the plant microbiome to improve drought tolerance: mechanisms and perspectives. Environ. Exp. Bot. 201:104933. doi: 10.1016/j.envexpbot.2022.104933

[ref11] Alok D. Annapragada H. Singh S. Ghosh P. Sengupta A. Basu D.. (2020). Symbiotic nitrogen fixation and endophytic bacterial community structure in Bt-transgenic chickpea (*Cicer arietinum* L). Sci. Rep. 10:5453. doi: 10.1038/s41598-020-62199-1 32214159 PMC7096491

[ref12] Anand G. Bhattacharjee A. Shrivas V. L. Dubey S. Sharma S. (2021). ACC deaminase positive Enterobacter-mediated mitigation of salinity stress, and plant growth promotion of *Cajanus cajan*: a lab to field study. Physiol. Mol. Biol. Plants 27, 1547–1557. doi: 10.1007/s12298-021-01031-0, PMID: 34366596 PMC8295421

[ref13] Ansari F. A. Ahmad I. Pichtel J. (2019). Growth stimulation and alleviation of salinity stress to wheat by the biofilm forming *Bacillus pumilus* strain FAB10. Appl. Soil Ecol. 143, 45–54. doi: 10.1016/j.apsoil.2019.05.023

[ref14] Anzano A. Bonanomi G. Mazzoleni S. Lanzotti V. (2022). Plant metabolomics in biotic and abiotic stress: a critical overview. Phytochem. Rev. 21, 503–524. doi: 10.1007/s11101-021-09786-w

[ref15] Aroca R. Luiz-Lozano J. M. Zamarreño Á. M. Paz J. A. García-Mina J. M. Pozo M. J.. (2013). Arbuscular mycorrhizal symbiosis influences strigolactone production under salinity and alleviates salt stress in lettuce plants. J. Plant Physiol. 170, 47–55. doi: 10.1016/j.jplph.2012.08.020 23102876

[ref16] Arun K. D. Sabarinathan K. G. Gomathy M. Kannan R. Balachandar D. (2020). Mitigation of drought stress in rice crop with plant growth-promoting abiotic stress-tolerant rice phyllosphere bacteria. J. Basic Microbiol. 60, 768–786. doi: 10.1002/jobm.202000011, PMID: 32667057

[ref17] Ashrafi E. Zahedi M. Razmjoo J. (2014). Co-inoculations of arbuscular mycorrhizal fungi and rhizobia under salinity in alfalfa. Soil Sci. Plant Nutr. 60, 619–629. doi: 10.1080/00380768.2014.936037

[ref18] Aslam M. M. Waseem M. Jakada B. H. Okal E. J. Lei Z. Sohaib H.. (2022). Mechanisms of abscisic acid-mdiated drought stress responses in plants. Int. J.Mol Sci. 19, 23:1084. doi: 10.3390/ijms23031084, PMID: 35163008 PMC8835272

[ref19] Avilés-García M. E. Flores-Cortez I. Hernández-Soberano C. Santoyo G. Valencia-Cantero E. (2016). La rizobacteria promotora del crecimiento vegeta: *Arthrobacter agilis* UMCV2 coloniza endofíticamente a *Medicago truncatula*. Rev. Argent. Microbiol. 48, 342–346. doi: 10.1016/j.ram.2016.07.004, PMID: 27916328

[ref20] Awasthy S. Kumar S. R. Sivakumar U. (2017). Mitigation of drought in rice by a phyllosphere bacterium *Bacillus altitudinis FD48*. Afr. J. Microbiol. Res. 11, 1614–1625. doi: 10.5897/AJMR2017.8610

[ref21] Ayangbenro A. S. Babalola O. O. (2017). A new strategy for heavy metal polluted environments: a review of microbial biosorbents. Int. J. Environ. Res. Public Health 14:94. doi: 10.3390/ijerph14010094, PMID: 28106848 PMC5295344

[ref22] Backer R. Rokem J. S. Ilangumaran G. Lamont J. Praslickova D. Ricci E.. (2018). Plant growth-promoting rhizobacteria: context, mechanisms of action, and roadmap to commercialization of biostimulants for sustainable agriculture. Front. Plant Sci. 9:1473. doi: 10.3389/fpls.2018.01473 30405652 PMC6206271

[ref23] Baltruschat H. Fodor J. Harrach B. D. Niemczyk E. Barna B. Gullner G.. (2008). Salt tolerance of barley induced by the root endophyte *Piriformospora indica* is associated with a strong increase in anti-oxidants. New Phytol. 180, 501–510. doi: 10.1111/j.1469-8137.2008.02583.x, PMID: 18681935

[ref24] Barea J. M. (2015). Future challenges and perspectives for applying microbial biotechnology in sustainable agriculture based on a better understanding of plant-microbiome interactions. J. Soil Sci. and Plant Nutr. 15, 261–282. doi: 10.4067/S0718-95162015005000021

[ref25] Batool T. Ali S. Seleiman M. F. Naveed N. H. Ali A. Ahmed K.. (2020). Plant growth promoting rhizobacteria alleviates drought stress in potato in response to suppressive oxidative stress and antioxidant enzymes activities. Sci. Rep. 10:16975. doi: 10.1038/s41598-020-73489-z 33046721 PMC7550571

[ref26] Bedano J. C. Vaquero F. Domínguez A. Rodríguez M. P. Wall L. Lavelle P. (2019). Earthworms contribute to ecosystem process in no-till Systems with high crop rotation intensity in Argentina. Acta Oecol. 98, 14–24. doi: 10.1016/j.actao.2019.05.003

[ref27] Begcy K. Sandhu J. Walia H. (2018). Transient heat stress during early seed development primes germination and seedling establishment in rice. Front. Plant Sci. 9:1768. doi: 10.3389/fpls.2018.01768 30568666 PMC6290647

[ref28] Begum Y. (2022). Regulatory role of microRNAs (miRNAs) in the recent development of abiotic stress tolerance of plants. Gene 821:146283. doi: 10.1016/j.gene.2022.146283, PMID: 35143944

[ref29] Belimov A. A. Dodd I. C. Hontzeas N. Theobald J. C. Safronova V. I. Davies W. J. (2009). Rhizosphere bacteria containing 1-aminocyclopropane1-carboxylate deaminase increase yield of plants grown in drying soil via both local and systemic hormone signalling. New Phytol. 181, 413–423. doi: 10.1111/j.1469-8137.2008.02657.x 19121036

[ref30] Ben Mahmoud O. M. Hidri R. Talbi-Zribi O. Taamalli W. Abdelly C. Djébali N. (2020). Auxin and proline producing rhizobacteria mitigate salt-induced growth inhibition of barley plants by enhancing water and nutrient status. S. Afr. J. Bot. 128, 209–217. doi: 10.1016/j.sajb.2019.10.023

[ref31] Benidire L. El Khalloufi F. Oufdou K. Barakat M. Tulumello J. Ortet P.. (2020). Phytobeneficial bacteria improve saline stress tolerance in *Vicia faba* and modulate microbial interaction network. Sci. Total Environ. 729:139020. doi: 10.1016/j.scitotenv.2020.139020 32498175

[ref32] Bernardo L. Carletti P. Badeck F. W. Rizza F. Morcia C. Ghizzoni R.. (2019). Metabolomic responses triggered by arbuscular mycorrhiza enhance tolerance to water stress in wheat cultivars. Plant Physiol. Biochem. 137, 203–212. doi: 10.1016/j.plaphy.2019.02.007, PMID: 30802803

[ref33] Brasileiro A. C. M. Morgante C. V. Araujo A. C. G. Leal-Bertioli S. C. M. Silva A. K. Martins A. C. Q.. (2015). Transcriptome profiling of wild Arachis from water-limited environments uncovers drought tolerance candidate genes. Plant Mol. Biol. Rep. 33, 1876–1892. doi: 10.1007/s11105-015-0882-x, PMID: 26752807 PMC4695501

[ref34] Brotman Y. Landau U. Cuadros-Inostroza Á. Takayuki T. Fernie A. R. Chet I.. (2013). Trichoderma-plant root colonization: escaping early plant defense responses and activation of the antioxidant machinery for saline stress tolerance. PLoS Pathog. 9:e1003221. doi: 10.1371/journal.ppat.1003221 23516362 PMC3597500

[ref35] Bruno L. B. Karthik C. Ma Y. Kadirvelu K. Freitas H. Rajkumar M. (2020). Amelioration of chromium and heat stresses in *Sorghum bicolor* by Cr(6þ) reducing thermos tolerant plant growth promoting bacteria. Chemosphere 244:125521. doi: 10.1016/j.chemosphere.2019.125521, PMID: 31812764

[ref36] Cabral C. Sabine R. Ivanka T. Bernd W. (2016). Arbuscular mycorrhizal fungi modify nutrient allocation and composition in wheat (*Triticum aestivum* L.) subjected to heat-stress. Plant Soil 408, 385–399. doi: 10.1007/s11104-016-2942-x

[ref37] Chandrasekaran M. Boopathi T. Manivannan P. (2021). Comprehensive assessment of ameliorative effects of AMF in alleviating abiotic stress in tomato plants. J Fungi 7:303. doi: 10.3390/jof7040303, PMID: 33921098 PMC8071382

[ref38] Chang P. Gerhardt K. E. Huang X.-D. Yu X.-M. Glick B. R. Gerwing P. D.. (2014). Plant growthpromoting bacteria facilitate the growth of barley and oats in saltimpacted soil: implications for phytoremediation of saline soils. Int. J. Phytoremediation 16, 1133–1147. doi: 10.1080/15226514.2013.821447, PMID: 24933907

[ref39] Chen L. Schwier M. Krumbach J. Kopriva S. Jacoby R. P. (2021). Metabolomics in plant-microbe interactions in the roots. Adv. Bot. Res. 98, 133–161. doi: 10.1016/bs.abr.2020.09.018

[ref40] Chen G. Zheng D. Feng N. Zhou H. Mu D. Zhao L.. (2022). Physiological mechanisms of ABA-induced salinity tolerance in leaves and roots of rice. Sci. Rep. 12:8228. doi: 10.1038/s41598-022-11408-0 35581217 PMC9114345

[ref41] Chialva M. Lanfranco L. Bonfante P. (2022). The plant microbiota: composition, functions, and engineering Curr. Opin. Biotechnol. 73, 135–142. doi: 10.1016/j.copbio.2021.07.003, PMID: 34392234

[ref42] Chihaoui S. A. Trabelsi D. Jdey A. Mhadhbi H. Mhamdi R. (2015). Inoculation of *Phaseolus vulgaris* with the nodule-endophyte *agrobacterium* sp. 10C2 affects richness and structure of rhizosphere bacterial communities and enhances nodulation and growth. Arch. Microbiol. 197, 805–813. doi: 10.1007/s00203-015-1118-z, PMID: 25967041

[ref43] Cho S. M. Kang B. R. Han S. H. Anderson A. J. Park J. Y. Lee Y. H.. (2008). 2R, 3R-butanediol, a bacterial volatile produced by *Pseudomonas chlororaphis* O6, is involved in induction of systemic tolerance to drought in *Arabidopsis thaliana*. Mol. Plant-Microbe Interact. 21, 1067–1075. doi: 10.1094/MPMI-21-8-1067 18616403

[ref44] Chukwuneme C. F. Babalola O. O. Kutu F. R. Ojuederie O. B. (2020). Characterization of actinomycetes isolates for plant growth promoting traits and their effects on drought tolerance in maize. J. Plant Interact. 15, 93–105. doi: 10.1080/17429145.2020.1752833

[ref45] Compant S. Marcel G. A. Heijden V. D. Sessitsch A. (2010). Climate change effects on beneficial plant–microorganism interactions. FEMS Microbiol. Ecol. 73, 197–214. doi: 10.1111/j.1574-6941.2010.00900.x, PMID: 20528987

[ref46] Crandall S. G. Gold K. M. Jiménez-Gasco M. D. M. Filgueiras C. C. Willett D. S. (2020). A multi-omics approach to solving problems in plant disease ecology. PLoS One 15:e0237975. doi: 10.1371/journal.pone.0237975, PMID: 32960892 PMC7508392

[ref47] Dardanelli M. S. Fernández de Córdoba F. J. Espuny M. R. Rodríguez Carvajal M. A. Soria Díaz M. E. Gil Serrano A. M.. (2008). Effect of *Azospirillum brasilense* coinoculated with rhizobium on *Phaseolus vulgaris* flavonoids and nod factor production under salt stress. Soil Biol. Biochem. 40, 2713–2721. doi: 10.1016/j.soilbio.2008.06.016

[ref48] Dastogeer K. M. G. Zahan M. I. Rhaman M. S. Sarker M. S. A. Chakraborty A. (2022). Microbe-mediated Thermotolerance in plants and pertinent mechanisms- a meta-analysis and review. Front. Microbiol. 13:511. doi: 10.3389/fmicb.2022.833566 PMC8940538 35330772

[ref49] Dawid C. Hille K. (2018). Functional metabolomics–a useful tool to characterize stress-induced Metabolome alterations opening new avenues towards tailoring food crop quality. Agronomy 8:138. doi: 10.3390/agronomy8080138

[ref50] Deokar A. A. Kondawar V. Jain P. K. Karuppayil M. Raju N. L. Vadez V.. (2011). Comparative analysis of expressed sequence tags (ESTs) between drought-tolerant and susceptible genotypes of chickpea under terminal drought stress. BMC Plant Biol. 11:70. doi: 10.1186/1471-2229-11-70 21513527 PMC3110109

[ref51] Dotaniya M. Saha J. (2016). Heavy metal polluted soils in India: status and countermeasures. JNKVV Res. J. 49, 320–337.

[ref52] Duc N. H. Csintalan Z. Posta K. (2018). Arbuscular mycorrhizal fungi mitigate negative effects of combined drought and heat stress on tomato plants. Plant Physiol. Biochem. 132, 297–307. doi: 10.1016/j.plaphy.2018.09.011 30245343

[ref53] El-Esawi M. A. Alaraidh I. A. Alsahli A. A. Alamri S. A. Ali H. M. Alayafi A. A. (2018). *Bacillus firmus* (SW5) augments salt tolerance in soybean (*Glycine max* L.) by modulating root system architecture, antioxidant defense systems and stress-responsive genes expression. Plant Physiol. Biochem. 132, 375–384. doi: 10.1016/j.plaphy.2018.09.026, PMID: 30268029

[ref54] El-Meihy R. Abou-Aly H. Youssef A. Tewfike T. Elakshar E. (2019). Efficiency of heavy metals-tolerant plant growth promoting bacteria for alleviating heavy metals toxicity on sorghum. Environ. Exp. Bot. 162, 295–301. doi: 10.1016/j.envexpbot.2019.03.005

[ref55] Escudero-Martinez C. Bulgarelli D. (2019). Tracing the evolutionary routes of plant-microbiota interactions. Curr. Opin. Microbiol. 49, 34–40. doi: 10.1016/j.mib.2019.09.013, PMID: 31698159

[ref56] Farooq M. Wahid A. Kobayashi N. Fujita D. Basra S. M. A. (2009). Plant drought stress: effects, mechanisms and management. Agron. Sustain. Dev. 29, 185–212. doi: 10.1051/agro:2008021, PMID: 37380788

[ref57] Farooqi M. Q. U. Nawaz G. Wani S. H. Choudhary J. R. Rana M. Sah R. P.. (2022). Recent developments in multi-omics and breeding strategies for abiotic stress tolerance in maize (*Zea mays* L.). Front. Plant Sci. 13:965878. doi: 10.3389/fpls.2022.965878, PMID: 36212378 PMC9538355

[ref58] Ferreira M. J. Silva H. Cunha A. (2019). Siderophore-producing rhizobacteria as a promising tool for empowering plants to cope with iron limitation in saline soils: a review. Pedosphere 29, 409–420. doi: 10.1016/S1002-0160(19)60810-6

[ref59] Friedrich T. Oberkofler V. Trindade I. Altmann S. Brzezinka K. Lämke J.. (2021). Heteromeric HSFA2/HSFA3 complexes drive transcriptional memory after heat stress in *Arabidopsis*. Nat. Commun. 12:23786. doi: 10.1038/s41467-021-23786-6, PMID: 34103516 PMC8187452

[ref60] Gangola M. P. Ramadoss B. R. (2020). “WRKY transcription factors for biotic and abiotic stress tolerance in plants” in Transcription factors for abiotic stress tolerance in plants. ed. Wani S. H. (Amsterdam: Elsevier), 15–28.

[ref61] Gentile A. Dias L. I. Mattos R. S. Ferreira T. H. Menossi M. (2015). MicroRNAs and drought responses in sugarcane. Front. Plant Sci. 6:58. doi: 10.3389/fpls.2015.00058, PMID: 25755657 PMC4337329

[ref62] Ghatak A. Chaturvedi P. Weckwerth W. (2018). Metabolomics in plant stress physiology. Adv. Biochem. Eng. Biotechnol. 164, 187–236. doi: 10.1007/10_2017_55, PMID: 29470599

[ref63] Ghorbanpour A. Salimi A. Ghanbary M. A. T. Pirdashti H. Dehestani A. J. S. H. (2018). The effect of *Trichoderma harzianum* in mitigating low temperature stress in tomato (*Solanum lycopersicum* L.) plants. Sci. Hortic. 230, 134–141. doi: 10.1016/j.scienta.2017.11.028

[ref64] Glick B. R. (2014). Bacteria with ACC deaminase can promote plant growth and help to feed the world. Microbiol. Res. 169, 30–39. doi: 10.1016/j.micres.2013.09.009, PMID: 24095256

[ref65] Goswami M. Deka S. (2020). Plant growth-promoting rhizobacteria-alleviators of abiotic stresses in soil: a review. Pedosphere 30, 40–61. doi: 10.1016/S1002-0160(19)60839-8

[ref66] Grover M. Bodhankar S. Maheswari M. Srinivasarao C. (2016). “Actinomycetes as mitigators of climate change and abiotic stress” in Plant growth promoting Actinobacteria. eds. Subramaniam G. Arumugam S. Rajendran V. (Springer: Singapore), 203–212.

[ref67] Haghighi M. Mozafariyan M. Abdolahipour B. (2015). Effect of cucumber mycorrhiza inoculation under low and high root temperature grown on hydroponic conditions. J. Crop. Sci. Biotechnol. 18, 89–96. doi: 10.1007/s12892-014-0083-4

[ref68] Hahm M. S. Son J. S. Hwang Y. J. Kwon D. K. Ghim S. Y. (2017). Alleviation of salt stress in pepper (*Capsicum annum* L.) plants by plant growth-promoting rhizobacteria. J. Microbiol. Biotechnol. 27, 1790–1797. doi: 10.4014/jmb.1609.09042, PMID: 28783895

[ref69] Hajiboland R. Joudmand A. Aliasgharzad N. Tolrá R. Poschenrieder C. (2019). Arbuscular mycorrhizal fungi alleviate low-temperature stress and increase freezing resistance as a substitute for acclimation treatment in barley. Crop Pasture Sci. 70, 218–233. doi: 10.1071/CP18385

[ref70] Hakim S. Naqqash T. Nawaz M. S. Laraib I. Siddique M. J. Zia R.. (2021). Rhizosphere engineering with plant growth-promoting microorganisms for agriculture and ecological sustainability. Front. Sustain. Food Syst. 5:617157. doi: 10.3389/fsufs.2021.617157, PMID: 36453930

[ref71] Haraguchi S. Yoshiga T. (2020). Potential of the fungal feeding nematode *Aphelenchus avenae* to control fungi and the plant parasitic nematode *Ditylenchus* destructor associated with garlic. Biol. Control 143:104203. doi: 10.1016/j.biocontrol.2020.104203

[ref72] Harun-Or-Rashid M. Chung Y. R. (2017). Induction of systemic resistance against insect herbivores in plants by beneficial soil microbes. Front. Plant Sci. 8:816. doi: 10.3389/fpls.2017.01816 29104585 PMC5654954

[ref73] Hasanuzzaman M. Bhuyan M. H. M. B. Zulfiqar F. Raza A. Mohsin S. M. Al Mahmud J.. (2020). Reactive oxygen species and antioxidant defense in plants under abiotic stress: revisiting the crucial role of a universal defense regulator. Antioxidants 9, 1–52. doi: 10.3390/antiox9080681 PMC7465626 32751256

[ref74] Hasanuzzaman M. Fujita M. (2022). Plant responses and tolerance to salt stress: physiological and molecular interventions. Int. J. Mol. Sci. 23:4810. doi: 10.3390/ijms23094810, PMID: 35563198 PMC9103774

[ref75] Hasanuzzaman M. Nahar K. Alam M. M. Roychowdhury R. Fujita M. (2013). Physiological, biochemical, and molecular mechanisms of heat stress tolerance in plants. Int. J. Mol. Sci. 14, 9643–9684. doi: 10.3390/ijms14059643, PMID: 23644891 PMC3676804

[ref76] Hashem A. Alqarawi A. A. Radhakrishnan R. Al-Arjani A. F. Aldehaish H. A. Egamberdieva D.. (2018). Arbuscular mycorrhizal fungi regulate the oxidative system, hormones and ionic equilibrium to trigger salt stress tolerance in *Cucumis sativus* L. Saudi J. Biol. Sci. 25, 1102–1114. doi: 10.1016/j.sjbs.2018.03.009, PMID: 30174509 PMC6117372

[ref77] Hassan M. Chattha M. Khan I. Chattha M. Barbanti L. Aamer M.. (2020). Heat stress in cultivated plants: nature, impact, mechanisms, and mitigation strategies - a review. Plant Biosyst. 155, 1–56. doi: 10.1080/11263504.2020.1727987

[ref78] Hayat R. Ali S. Amara U. Ali U. Amara R. Khalid I.. (2010). Soil beneficial bacteria and their role in plant growth promotion: a review. Ann. Microbiol. 60, 579–598. doi: 10.1007/s13213-010-0117-1

[ref79] Hünninghaus M. Koller R. Kramer S. Marhan S. Kandeler E. Bonkowski M. (2017). Changes in bacterial community composition and soil respiration indicate rapid successions of protist grazers during mineralization of maize crop residues. Pedobiologia 62, 1–8. doi: 10.1016/j.pedobi.2017.03.002

[ref80] Iqbal S. Wang X. Mubeen I. Kamran M. Kanwal I. Díaz G. A.. (2022). Phytohormones trigger drought tolerance in crop plants: outlook and future perspectives. Front. Plant Sci. 12:799318. doi: 10.3389/fpls.2021.799318, PMID: 35095971 PMC8792739

[ref81] Islam F. Yasmeen T. Ali S. Ali D. Hameed S. Zhou W.. (2016). Plant growth promoting bacteria confer salt tolerance in *Vigna radiata* by up-regulating antioxidant defense and biological soil fertility. Plant Growth Regul. 80, 23–26. doi: 10.1007/s10725-015-0142-y

[ref82] Jaber L. R. Alananbeh K. M. (2018). Fungal entomopathogens as endophytes reduce several species of Fusarium causing crown and root rot in sweet pepper (*Capsicum annuum* L.). Biol. Control 126, 117–126. doi: 10.1016/j.biocontrol.2018.08.007

[ref83] Jacob P. Hirt H. Bendahmane A. (2017). The heat-shock protein/ chaperone network and multiple stress resistance. Plant Biotechnol. J. 15, 405–414. doi: 10.1111/pbi.12659, PMID: 27860233 PMC5362687

[ref84] Janiak A. Kwaśniewsk M. Szarejko I. (2016). Gene expression regulation in roots under drought. J. Exp. Bot. 67, 1003–1014. doi: 10.1093/jxb/erv512, PMID: 26663562

[ref85] Javaid M. M. Florentine S. Mahmood A. Wasaya A. Javed T. Sattar A.. (2022). Interactive effect of elevated CO_2_ and drought on physiological traits of *Datura stramonium*. Front. Plant Sci. 13:929378. doi: 10.3389/fpls.2022.929378, PMID: 36388510 PMC9644026

[ref86] Jeong D. H. Park S. Zhai J. Gurazada S. G. De Paoli E. Meyers B. C.. (2011). Massive analysis of rice small RNAs: mechanistic implications of regulated microRNAs and variants for differential target RNA cleavage. Plant Cell 23, 4185–4207. doi: 10.1105/tpc.111.089045, PMID: 22158467 PMC3269859

[ref87] Jha U. C. Bohra A. Jha R. Parida S. K. (2019). Salinity stress response and ‘omics’ approaches for improving salinity stress tolerance in major grain legumes. Plant Cell Rep. 38, 255–277. doi: 10.1007/s00299-019-02374-5, PMID: 30637478

[ref88] Jha Y. Mohamed H. I. (2022). Inoculation with *Lysinibacillus fusiformis* strain YJ4 and *Lysinibacillus sphaericus* strain YJ5 alleviates the effects of cold stress in maize plants. Gesunde Pflanz. 75, 77–95. doi: 10.1007/s10343-022-00666-7

[ref89] Jiang Q. Y. Zhuo F. Long S. H. Zhao H. D. Yang D. J. Ye Z. H.. (2016). Can arbuscular mycorrhizal fungi reduce cd uptake and alleviate cd toxicity of *Lonicera japonica* grown in cd-added soils? Sci. Rep. 6:21805. doi: 10.1038/srep21805 26892768 PMC4759589

[ref90] Jin P. Tan Z. Wang H. Liu W. Miao W. (2021). Antimicrobial effect of *Bacillus licheniformis HN-5* bacitracin a on rice pathogen *Pantoea ananatis*. Biol. Control 66, 249–257. doi: 10.1007/s10526-020-10052-9

[ref91] Johnston-Monje D. Raizada M. N. (2011). Conservation and diversity of seed associated endophytes in Zea across boundaries of evolution, ethnography and ecology. PLoS One 6:e20396. doi: 10.1371/journal.pone.0020396, PMID: 21673982 PMC3108599

[ref92] Joshi H. Shourie A. Singh A. (2020). Cyanobacteria as a source of biofertilizers for sustainable agriculture. In Singh P. K. Kumar A. Singh V. K. Shrivistava A. K., Advances in cyanobacterial biology (pp. 385–396). Cambrigge, MA: Academic Press.

[ref93] Kakar K. Ren X. L. Nawaz Z. Cui Z. Li B. Xie G.. (2015). Consortium of Rhizobacterial strains and biochemical growth elicitors improve cold and drought stress tolerance in rice (*Oryza Sativa* L.). Plant Biol. 3, 471–483. doi: 10.1111/plb.12427 26681628

[ref94] Karthik C. Oves M. Thangabalu R. Sharma R. Santhosh S. B. Indra Arulselvi P. (2016). *Cellulosimicrobium funkei*-like enhances the growth of *Phaseolus vulgaris* by modulating oxidative damage under chromium (VI) toxicity. J. Adv. Res. 7, 839–850. doi: 10.1016/j.jare.2016.08.007, PMID: 27668092 PMC5026708

[ref95] Katam R. Lin C. Grant K. Katam C. S. Chen S. (2022). Advances in plant metabolomics and its applications in stress and single-cell biology. Int. J. Mol. Sci. 23:6985. doi: 10.3390/ijms23136985, PMID: 35805979 PMC9266571

[ref96] Katano K. Honda K. Suzuki N. (2018). Integration between ROS regulatory systems and other signals in the regulation of various types of heat responses in plants. Int. J. Mol. Sci. 19:3370. doi: 10.3390/ijms19113370, PMID: 30373292 PMC6274784

[ref97] Kaul S. Choudhary M. Gupta S. Dhar M. K. (2021). Engineering host microbiome for crop improvement and sustainable agriculture. Front. Microbiol. 28:635917. doi: 10.3389/fmicb.2021.635917 PMC8193672 34122359

[ref98] Kaushal M. Wani S. P. (2016). Plant-growth-promoting rhizobacteria: drought stress alleviators to ameliorate crop production in drylands. Ann. Microbiol. 66, 35–42. doi: 10.1007/s13213-015-1112-3

[ref99] Keyster M. Niekerk L. A. Basson G. Carelse M. Bakare O. Ludidi N.. (2020). Decoding heavy metal stress signalling in plants: towards improved food security and safety. Plan. Theory 9, 1–26. doi: 10.3390/plants9121781 PMC7765602 33339160

[ref100] Khan M. A. Asaf S. Khan A. L. Adhikari A. Jan R. Ali S.. (2020). Plant growth-promoting endophytic bacteria augment growth and salinity tolerance in rice plants. Plant Biol. 22, 850–862. doi: 10.1111/plb.13124, PMID: 32329163

[ref101] Khan M. A. Asaf S. Khan A. L. Ullah I. Ali S. Kang S. M.. (2019). Alleviation of salt stress response in soybean plants with the endophytic bacterial isolate *Curtobacterium* sp. SAK1. Ann. Microbiol. 69, 797–808. doi: 10.1007/s13213-019-01470-x

[ref102] Khan A. L. Kang S. M. Dhakal K. H. Hussain J. Adnan M. Kim J. G.. (2013). Flavonoids and amino acid regulation in *Capsicum annuum* L. by endophytic fungi under different heat stress regimes. Sci. Hortic. 155, 1–7. doi: 10.1016/j.scienta.2013.02.028

[ref103] Khan A. Khan V. Pandey K. Sopory S. K. Sanan-Mishra N. (2022). Thermo-priming mediated cellular networks for abiotic stress management in plants. Front. Plant Sci. 13:866409. doi: 10.3389/fpls.2022.866409, PMID: 35646001 PMC9136941

[ref104] Khan A. Sirajuddin Z. Zhao X. Q. Javed M. T. Khan K. S. Bano A.. (2016). *Bacillus pumilus* enhances tolerance in rice (*Oryza sativa* L.) to combined stresses of NaCl and high boron due to limited uptake of Na+. Environ. Exp. Bot. 124, 120–129. doi: 10.1016/j.envexpbot.2015.12.011

[ref105] Khatabi B. Gharechahi J. Ghaffari M. R. Liu D. Haynes P. A. McKay M. J.. (2019). Plant-microbe symbiosis: what has proteomics taught us? Proteomics 19:e1800105. doi: 10.1002/pmic.201800105, PMID: 31218790

[ref106] Kissoudis C. Kalloniati C. Flemetakis E. Madesis P. Labrou N. E. Tsaftaris A.. (2015). Stress-inducible GmGSTU4 shapes transgenic tobacco plants metabolome towards increased salinity tolerance. Acta Physiol. Plant. 37, 1–11. doi: 10.1007/s11738-015-1852-5

[ref107] Köhl J. Medeiros F. H. Lombaers-van der Plas C. Groenenboom-de Haas L. van den Bosch T. (2020). Efficacies of bacterial and fungal isolates in biocontrol of *Botrytis cinerea* and *Pseudomonas syringae* pv. Tomato and growth promotion in tomato do not correlate. Biol. Control 150:104375. doi: 10.1016/j.biocontrol.2020.104375

[ref108] Kong A. Y. Y. Six J. (2012). Microbial community assimilation of cover crop rhizodeposition within soil microenvironments in alternative and conventional cropping systems. Plant Soil 356, 315–330. doi: 10.1007/s11104-011-1120-4

[ref109] Krasensky J. Jonak C. (2012). Drought, salt, and temperature stress-induced metabolic rearrangements and regulatory networks. J. Exp. Bot. 63, 1593–1608. doi: 10.1093/jxb/err460, PMID: 22291134 PMC4359903

[ref110] Krome K. Rosenberg K. Dickler C. Kreuzer K. Ludwig-Müller J. Ullrich-Eberius C.. (2010). Soil bacteria and protozoa affect root branching via effects on the Auxin and Cytokinin balance in plants. Plant Soil 328, 191–201. doi: 10.1007/s11104-009-0101-3

[ref111] Kumar P. Choudhary M. Halder T. Prakash N. R. Singh V. Sheoran S.. (2022). Salinity stress tolerance and omics approaches: revisiting the progress and achievements in major cereal crops. Heredity 128, 497–518. doi: 10.1038/s41437-022-00516-2 35249098 PMC9177680

[ref112] Kumar P. Sharma P. K. (2020). Soil salinity and food security in India. Front. Sustain. Food Syst. 4:533781. doi: 10.3389/fsufs.2020.533781, PMID: 37146830

[ref113] Kumar A. Singh S. Gaurav A. K. Srivastava S. Verma J. P. (2020). Plant growth-promoting bacteria: biological tools for the mitigation of salinity stress in plants. Front. Microbiol. 11:1216. doi: 10.3389/fmicb.2020.01216, PMID: 32733391 PMC7358356

[ref114] Kurniawan S. B. Ramli N. N. Said N. S. M. Alias J. Imron M. F. Abdullah S. R. S.. (2022). Practical limitations of bioaugmentation in treating heavy metal contaminated soil and role of plant growth promoting bacteria in phytoremediation as a promising alternative approach. Heliyon 8:e08995. doi: 10.1016/j.heliyon.2022.e08995, PMID: 35399376 PMC8983376

[ref115] Kuromori T. Fujita M. Takahashi F. Yamaguchi-Shinozaki K. Shinozaki K. (2022). Inter-tissue and inter-organ signaling in drought stress response and phenotyping of drought tolerance. Plant J. 109, 342–358. doi: 10.1111/tpj.15619, PMID: 34863007 PMC9300012

[ref116] Kuske C. R. Hesse C. N. Challacombe J. F. Cullen D. Herr J. R. Mueller R. C.. (2015). Prospects and challenges for fungal metatranscriptomics of complex communities. Fungal Ecol. 14, 133–137. doi: 10.1016/j.funeco.2014.12.005, PMID: 34693062

[ref117] Lareen A. Burton F. Schäfer P. (2016). Plant root-microbe communication in shaping root microbiomes. Plant Mol. Biol. 90, 575–587. doi: 10.1007/s11103-015-0417-8, PMID: 26729479 PMC4819777

[ref118] Lau E. T. Tani A. Khew C. Y. Chua Y. Q. San Hwang S. (2020). Plant growth-promoting bacteria as potential bio-inoculants and biocontrol agents to promote black pepper plant cultivation. Microbiol. Res. 240:126549. doi: 10.1016/j.micres.2020.126549, PMID: 32688172

[ref119] Lau S. E. Teo W. F. A. Teoh E. Y. Tan B. C. (2022). Microbiome engineering and plant biostimulants for sustainable crop improvement and mitigation of biotic and abiotic stresses. Discover Food 2:9. doi: 10.1007/s44187-022-00009-5 PMC9573444 36235491

[ref120] Le D. T. Nishiyama R. Watanabe Y. Tanaka M. Seki M. Ham L. H.. (2012). Differential gene expression in soybean leaf tissues at late developmental stages under drought stress revealed by genome-wide transcriptome analysis. PLoS One 7:e49522. doi: 10.1371/journal.pone.0049522, PMID: 23189148 PMC3505142

[ref121] Levy A. Conway J. M. Dangl J. L. Woyke T. (2018). Elucidating bacterial gene functions in the plant microbiome. Cell Host Microbe 24, 475–485. doi: 10.1016/j.chom.2018.09.005, PMID: 30308154

[ref123] Liu H. H. Tian X. Li Y. J. Wu C. A. Zheng C. C. (2008). Microarray-based analysis of stress-regulated microRNAs in *Arabidopsis thaliana*. RNA 14, 836–843. doi: 10.1261/rna.895308, PMID: 18356539 PMC2327369

[ref124] Lu X. Jin C. Yang J. Liu Q. Wu S. Li D.. (2013). Prenatal and lactational Lead exposure enhanced oxidative stress and altered apoptosis status in offspring rats’ hippocampus. Biol. Trace Elem. Res. 151, 75–84. doi: 10.1007/s12011-012-9531-5 23086308

[ref125] Lv D. K. Bai X. Li Y. Ding X. D. Ge Y. Cai H.. (2010). Profiling of cold-stress-responsive miRNAs in rice by microarrays. Gene 459, 39–47. doi: 10.1016/j.gene.2010.03.011, PMID: 20350593

[ref126] Ma Y. Rajkumar M. Rocha I. Oliveira R. S. Freitas H. (2015). Serpentine bacteria influence metal translocation and bioconcentration of *Brassica juncea* and *Ricinus communis* grown in multi-metal polluted soils. Front. Plant Sci. 5:757. doi: 10.3389/fpls.2014.00757 25601876 PMC4283507

[ref127] Magallon-Servin P. Antoun H. Taktek S. Taktek S. De-Bashan L. E. (2020). Designing a multi-species inoculant of phosphate rock-solubilizing bacteria compatible with arbuscular mycorrhizae for plant growth promotion in low-P soil amended with PR. Biol. Fertil. Soils 56, 521–536. doi: 10.1007/s00374-020-01452-1

[ref128] Mahmud K. Missaoui A. Lee K. Ghimire B. Presley H. W. Makaju S. (2021). Rhizosphere microbiome manipulation for sustainable crop production. Curr. Plant Biol. 27:100210. doi: 10.1016/j.cpb.2021.100210, PMID: 37222817

[ref129] Marcelino V. R. Irinyi L. Eden J. S. Meyer W. Holmes E. C. Sorrell T. C. (2019). Metatranscriptomics as a tool to identify fungal species and subspecies in mixed communities–a proof of concept under laboratory conditions. IMA Fungus. 10:12. doi: 10.1186/s43008-019-0012-8 32355612 PMC7184889

[ref130] Mathur S. Jajoo A. (2020). Arbuscular mycorrhizal fungi protects maize plants from high temperature stress by regulating photosystem II heterogeneity. Ind. Crop. Prod. 143:111934. doi: 10.1016/j.indcrop.2019.111934

[ref131] Meena K. K. Kumar M. Kalyuzhnaya M. G. Yandigeri M. S. Singh D. P. Saxena A. K.. (2012). Epiphytic pink-pigmented methylotrophic bacteria enhance germination and seedling growth of wheat (*Triticum aestivum*) by producing phytohormone. Anton. Van Leeuw. 101, 777–786. doi: 10.1007/s10482-011-9692-9 22200783

[ref132] Mehmood S. Khatoon Z. Ahmad I. A. Muneer M. A. Kamran M. A.. (2021). *Bacillus* sp. PM31 harboring various plant growth-promoting activities regulates *Fusarium* dry rot and wilt tolerance in potato. Arch. Agron. Soil Sci. 2021, 1–15. doi: 10.1080/03650340.2021.1971654

[ref133] Mitra S. Pramanik K. Sarkar A. Ghosh P. Soren T. Maiti T. (2018). Bioaccumulation of cadmium by *Enterobacter* sp. and enhancement of rice seedling growth under cadmium stress. Ecotoxicol. Environ. Saf. 156, 183–196. doi: 10.1016/j.ecoenv.2018.03.001, PMID: 29550436

[ref134] Mitter B. N. Pfaffenbichler R. Flavell S. Compant L. Antonielli A. Petric A.. (2017). A new approach to modify plant microbiomes and traits by introducing beneficial bacteria at flowering into progeny seeds. Front. Microbiol. 8:11. doi: 10.3389/fmicb.2017.00011, PMID: 28167932 PMC5253360

[ref135] Moldovan D. Spriggs A. Yang J. Pogson B. J. Dennis E. S. Wilson I. W. (2010). Hypoxia-responsive microRNAs and trans-acting small interfering RNAs in *Arabidopsis*. J. Exp. Bot. 61, 165–177. doi: 10.1093/jxb/erp296, PMID: 19815687 PMC2791121

[ref136] Mueller U. G. Sachs J. L. (2015). Engineering microbiomes to improve plant and animal health. Trends Microbiol. 23, 606–617. doi: 10.1016/j.tim.2015.07.009, PMID: 26422463

[ref137] Mukherjee P. Mitra A. Roy M. (2019). Halomonas rhizobacteria of *Avicennia marina* of Indian Sundarbans promote rice growth under saline and heavy metal stresses through exopolysaccharide production. Front. Microbiol. 10:1207. doi: 10.3389/fmicb.2019.01207 31191507 PMC6549542

[ref138] Mukhtar T. Ali F. Rafique M. Ali J. Afridi M. S. Smith D.. (2022). Biochemical characterization and potential of *Bacillus safensis* strain SCAL1 to mitigate heat stress in *Solanum lycopersicum* L. J. Plant Growth Regul. 139, 569–577. doi: 10.1007/s00344-021-10571-4

[ref139] Mukhtar T. Ur Rehman S. Smith D. Sultan T. Seleiman M. F. Alsadon A. A.. (2020). Mitigation of heat stress in *Solanum lycopersicum* L. by ACC-deaminase and exopolysaccharide producing *Bacillus cereus*: effects on biochemical profiling. Sustainability. 12:2159. doi: 10.3390/su12062159

[ref140] Mukhtar S. Zareen M. Khaliq Z. Mehnaz S. Malik K. A. (2020). Phylogenetic analysis of halophyte-associated rhizobacteria and effect of halotolerant and halophilic phosphate-solubilizing biofertilizers on maize growth under salinity stress conditions. J. Appl. Microbiol. 128, 556–573. doi: 10.1111/jam.14497 31652362

[ref141] Murali M. Singh S. B. Gowtham H. G. Shilpa N. Prasad M. Aiyaz M.. (2021). Induction of drought tolerance in *Pennisetum glaucum* by ACC deaminase producing PGPR- *Bacillus amyloliquefaciens* through antioxidant defence system. Microbiol. Res. 253:126891. doi: 10.1016/j.micres.2021.126891, PMID: 34656832

[ref142] Nadeem S. M. Zahir Z. A. Naveed M. Arshad M. (2007). Preliminary investigations on inducing salt tolerance in maize through inoculation with rhizobacteria containing ACC deaminase activity. Can. J. Microbiol. 53, 1141–1149. doi: 10.1139/W07-081, PMID: 18026206

[ref143] Naveed M. Hussain M. B. Zahir Z. A. Mitter B. Sessitsch A. (2014a). Drought stress amelioration in wheat through inoculation with *Burkholderia phytofirmans* strain *PsJN*. Plant Growth Regul. 73, 121–131. doi: 10.1007/s10725-013-9874-8

[ref144] Naveed M. Mitter B. Reichenauer T. G. Wieczorek K. Sessitsch A. (2014b). Increased drought stress resilience of maize through endophytic colonization by *Burkholderia phytofirmans PsJN* and *Enterobacter* sp FD17. Environ. Exp. Bot. 97, 30–39. doi: 10.1016/j.envexpbot.2013.09.014

[ref145] Naveed M. Mustafa A. Majeed S. Naseem Z. Saeed Q. Khan A.. (2020). Enhancing cadmium tolerance and pea plant health through *Enterobacter sp. MN17* inoculation together with biochar and gravel sand. Plan. Theory 9:530. doi: 10.3390/plants9040530, PMID: 32326023 PMC7238170

[ref146] Nazli F. Mustafa A. Ahmad M. Hussain A. Jamil M. Wang X.. (2020). A review on practical application and potentials of phytohormone-producing plant growthpromoting rhizobacteria for inducing heavy metal tolerance in crops. Sustainability 12:9056. doi: 10.3390/su12219056

[ref147] Nelson D. E. Repetti P. P. Adams T. R. Creelman R. A. Wu J. Warner D. C.. (2007). Plant nuclear factor Y (NF-Y) B subunits confer drought tolerance and lead to improved corn yields on water-limited acres. Proc. Natl. Acad. Sci. U. S. A. 104, 16450–16455. doi: 10.1073/pnas.0707193104, PMID: 17923671 PMC2034233

[ref148] Ngumbi E. Kloepper J. (2016). Bacterial-mediated drought tolerance: current and future prospects. Appl. Soil Ecol. 105, 109–125. doi: 10.1016/j.apsoil.2016.04.009

[ref149] Nilsson R. H. Anslan S. Bahram M. Wurzbacher C. Baldrian P. Tedersoo L. (2019). Mycobiome diversity: high-throughput sequencing and identification of fungi. Nat. Rev. Microbiol. 17, 95–109. doi: 10.1038/s41579-018-0116-y, PMID: 30442909

[ref150] Oliveira C. A. Alves V. M. C. Marriel I. E. Gomes E. A. Scotti M. R. Carneiro N. P.. (2009). Phosphate solubilizing microorganisms isolated from rhizosphere of maize cultivated in an oxisol of the Brazilian Cerrado biome. Soil Biol. Biochem. 41, 1782–1787. doi: 10.1016/j.soilbio.2008.01.012

[ref151] Omar M. N. A. Osman M. E. H. Kasim W. A. Abd El-Daim I. A. (2009). Improvement of salt tolerance mechanisms of barley cultivated under salt stress using *Azospirillum brasiliense*. Tasks Veg. Sci. 44, 133–147. doi: 10.1007/978-1-4020-9065-3_15

[ref152] Omer A. M. Emara H. M. Zaghloul R. A. Abdel-Monem M. O. Dawwam G. E. (2016). Potential of *Azotobacter salinestris* as plant growth promoting rhizobacteria under saline stress conditions. Res. J. Pharm. Biol. Chem. Sci. 7, 2572–2583.

[ref153] Orozco-Mosqueda M. D. C. Rocha-Granados M. D. C. Bernard R. G. Santoyo G. (2018). Microbiome engineering to improve biocontrol and plant growth-promoting mechanisms. Microbiol. Res. 208, 25–31. doi: 10.1016/j.micres.2018.01.005 29551209

[ref154] Ortiz N. Armadaa E. Duque E. Roldánc A. Azcóna R. (2015). Contribution of arbuscular mycorrhizal fungi and/or bacteria to enhancing plant drought tolerance under natural soil conditions: effectiveness of autochthonous or allochthonous strains. J. Plant Physiol. 174, 87–96. doi: 10.1016/j.jplph.2014.08.019, PMID: 25462971

[ref155] Oubohssaine M. Sbabou L. Aurag J. (2022). Native heavy metal-tolerant plant growth promoting rhizobacteria improves *Sulla spinosissima* (L.) growth in post-mining contaminated soils. Microorganisms. 10:50838. doi: 10.3390/microorganisms10050838, PMID: 35630284 PMC9144414

[ref156] Pal K. K. Dey R. Sherathia D. N. Devidayal M. Mangalassery S. Kumar A.. (2021). Alleviation of salinity stress in peanut by application of endophytic bacteria. Front. Microbiol. 12:650771. doi: 10.3389/fmicb.2021.650771, PMID: 33936008 PMC8079962

[ref157] Palaniyandi S. A. Damodharan K. Yang S. H. Suh J. W. (2014). *Streptomyces* sp. strain *PGPA39* alleviates salt stress and promotes growth of ‘micro tom’ tomato plants. J. Appl. Microbiol. 117, 766–773. doi: 10.1111/jam.12563 24909841

[ref158] Pandey V. Ansari M. W. Tula S. Yadav S. Sahoo R. K. Shukla N.. (2016). Dose-dependent response of *Trichoderma harzianum* in improving drought tolerance in rice genotypes. Planta 243, 1251–1264. doi: 10.1007/s00425-016-2482-x 26898554

[ref159] Pandey A. K. Rubiales D. Wang Y. Fang P. Sun T. Liu N.. (2021). Omics resources and omics-enabled approaches for achieving high productivity and improved quality in pea (*Pisum Sativum* L.). Theor. Appl. Genet. 134, 755–776. doi: 10.1007/s00122-020-03751-5, PMID: 33433637

[ref160] Panke-Buisse K. Poole A. C. Goodrich J. K. Ley R. E. Kao-Kniffin J. (2015). Selection on soil microbiomes reveals reproducible impacts on plant function. ISME J. 9, 980–989. doi: 10.1038/ismej.2014.196, PMID: 25350154 PMC4817706

[ref161] Panlada T. Pongdet P. Aphakorn L. Rujirek N.-N. Nantakorn B. Neung T. (2013). Alleviation of the effect of environmental stresses using co-inoculation of mungbean by *Bradyrhizobium* and rhizobacteria containing stress-induced ACC deaminase enzyme. Soil Sci. Plant Nutr. 59, 559–571. doi: 10.1080/00380768.2013.804391

[ref162] Parnell J. J. Berka R. Young H. A. Sturino J. M. Kang Y. Barnhart D. M.. (2016). From the lab to the farm: an industrial perspective of plant beneficial microorganisms. Front. Plant Sci. 7:1110. doi: 10.3389/fpls.2016.01110, PMID: 27540383 PMC4973397

[ref163] Pascale A. Proietti S. Pantelides I. S. Stringlis I. A. (2020). Modulation of the root microbiome by plant molecules: the basis for targeted disease suppression and plant growth promotion. Front. Plant Sci. 2020:1741. doi: 10.3389/fpls.2019.01741 PMC6992662 32038698

[ref164] Pavlova A. S. Leontieva M. R. Smirnova T. A. Kolomeitseva G. L. Netrusov A. I. Tsavkelova E. A. (2017). Colonization strategy of the endophytic plant growth promoting strains of *Pseudomonas fluorescens* and *Klebsiella oxytoca* on the seeds, seedlings and roots of the epiphytic orchid, *Dendrobium nobile* Lindl. J. Appl. Microbiol. 123, 217–232. doi: 10.1111/jam.13481, PMID: 28457004

[ref165] Pinedo I. Ledger T. Greve M. Poupin M. J. (2015). *Burkholderia phytofirmans PsJN* induces long-term metabolic and transcriptional changes involved in *Arabidopsis thaliana* salt tolerance. Front. Plant Sci. 6:466. doi: 10.3389/fpls.2015.00466, PMID: 26157451 PMC4477060

[ref166] Pramanik K. Mitra S. Sarkar A. Soren T. Maiti T. K. (2017). Characterization of cadmium-resistant *Klebsiella pneumoniae MCC 3091* promoted rice seedling growth by alleviating phytotoxicity of cadmium. Environ. Sci. Pollut. Control Ser. 24, 24419–24437. doi: 10.1007/s11356-017-0033-z 28895046

[ref167] Rani B. A. Madan S. Pooja K. S. Sharma K. D. Kumari N. Kumar A. (2018). Mitigating the effect of drought stress on yield in wheat (*Triticum aestivum*) using arbuscular mycorrhiza fungi (*Glomus mosseae*). Indian J. Agric. Sci. 88, 95–100. doi: 10.56093/ijas.v88i12.85444

[ref168] Raymond J. Siefart J. L. Staples C. R. Blakenship R. E. (2004). The natural history of nitrogen fixation. Mol. Biol. Evol. 21, 541–554. doi: 10.1093/molbev/msh047 14694078

[ref169] Richter J. A. Erban A. Kopka J. Zörb C. (2015). Metabolic contribution to salt stress in two maize hybrids with contrasting resistance. Plant Sci. 233, 107–115. doi: 10.1016/j.plantsci.2015.01.006, PMID: 25711818

[ref170] Rivero J. Álvarez D. Flors V. Azcón-Aguilar C. Pozo M. J. (2018). Root metabolic plasticity underlies functional diversity in mycorrhiza-enhanced stress tolerance in tomato. New Phytol. 220, 1322–1336. doi: 10.1111/nph.15295, PMID: 29982997

[ref171] Rodríguez M. Torres M. Blanco L. Béjar V. Sampedro I. Llamas I. (2020). Plant growth-promoting activity and quorum quenching-mediated biocontrol of bacterial phytopathogens by *Pseudomonas segetis* strain *P6*. Sci. Rep. 10:4121. doi: 10.1038/s41598-020-61084-1 32139754 PMC7058018

[ref172] Rojas-Solís D. Zetter-Salmón E. Contreras-Pérez M. del Carmen Rocha-Granados M. Macías-Rodríguez L. Santoyo G. (2018). *Pseudomonas stutzeri* E25 and *Stenotrophomonas maltophilia* CR71 endophytes produce antifungal volatile organic compounds and exhibit additive plant growth-promoting effects. Biocatal. Agric. Biotechnol. 13, 46–52. doi: 10.1016/j.bcab.2017.11.007

[ref173] Rojas-Tapias D. Moreno-Galvan A. Pardo-Diaz S. Obando M. Riveria D. Bonilla R. (2012). Effect of inoculation with plant growth-promoting bacteria (PGPB) on amelioration of saline stress in maize (*Zea mays*). Appl. Soil Ecol. 61, 264–272. doi: 10.1016/j.apsoil.2012.01.006

[ref174] Saeed Q. Xiukang W. Haider F. U. Kučerik J. Mumtaz M. Z. Holatko J.. (2021). Rhizosphere bacteria in plant growth promotion, biocontrol, and bioremediation of contaminated sites: a comprehensive review of effects and mechanisms. Int. J. Mol. Sci. 22:10529. doi: 10.3390/ijms221910529, PMID: 34638870 PMC8509026

[ref175] Sagar A. Rathore P. Ramteke P. W. Ramakrishna W. Reddy M. S. Pecoraro L. (2021). Plant growth promoting rhizobacteria, arbuscular mycorrhizal fungi and their synergistic interactions to counteract the negative effects of saline soil on agriculture: key macromolecules and mechanisms. Microorganisms 9:1491. doi: 10.3390/microorganisms9071491, PMID: 34361927 PMC8307984

[ref176] Sahoo R. K. Ansari M. W. Dangar T. K. Mohanty S. Tuteja N. (2014). Phenotypic and molecular characterisation of efficient nitrogen-fixing Azotobacter strains from rice fields for crop improvement. Protoplasma 251, 511–523. doi: 10.1007/s00709-013-0547-2, PMID: 24005473

[ref177] Salam U. Ullah S. Tang Z. H. Elateeq A. A. Khan Y. Khan J.. (2023). Plant metabolomics: an overview of the role of primary and secondary metabolites against different environmental stress factors. Life 13:706. doi: 10.3390/life13030706, PMID: 36983860 PMC10051737

[ref178] Sandrini M. Nerva L. Sillo F. Balestrini R. Chitarra W. Zampieri E. (2022). Abiotic stress and belowground microbiome: the potential of omics approaches. Int. J. Mol. Sci. 23:1091. doi: 10.3390/ijms23031091 35163015 PMC8835006

[ref179] Santos L. F. Olivares F. L. (2021). Plant microbiome structure and benefits for sustainable agriculture. Current Plant Biology. 26:100198. doi: 10.1016/j.cpb.2021.100198, PMID: 36998097

[ref180] Santoyo G. (2022). How plants recruit their microbiome? New insights into beneficial interactions. J. Adv. Res. 40, 45–58. doi: 10.1016/j.jare.2021.11.020, PMID: 36100333 PMC9481936

[ref181] Sehar Z. Gautam H. Iqbal N. Alvi A. F. Jahan B. Fatma M.. (2022). The functional interplay between ethylene, hydrogen sulfide, and sulfur in plant heat stress tolerance. Biomol. Ther. 12:678. doi: 10.3390/biom12050678, PMID: 35625606 PMC9138313

[ref182] Sen S. Chandrasekhar C. N. (2014). Effect of PGPR on growth promotion of rice (*Oryza sativa* L.) under salt stress. Asian J. Plant Sci. Res. 4, 62–67.

[ref183] Sexton W. K. Fidero M. Spain J. C. Jiang L. Bucalo K. Cruse-Sanders J. M.. (2019). Characterization of endophytic bacterial communities within greenhouse and field-grown rhizomes of three rare pitcher plant species (*Sarracenia oreophila*, *S. leucophylla*, and *S. purpurea* spp. *venosa*) with an emphasis on nitrogen-fixing bacteria. Plant Soil 447, 257–279. doi: 10.1007/s11104-019-04372-8

[ref184] Shaffique S. Khan M. A. Imran M. Kang S. M. Park Y. S. Wani S. H.. (2022). Research progress in the field of microbial mitigation of drought stress in plants. Front. Plant Sci. 13:870626. doi: 10.3389/fpls.2022.870626, PMID: 35665140 PMC9161204

[ref185] Sharma M. Sudheer S. Usmani Z. Rani R. Gupta P. (2020). Deciphering the omics of plant-microbe interaction: perspectives and new insights. Curr. Genomics 21, 343–362. doi: 10.2174/1389202921999200515140420, PMID: 33093798 PMC7536805

[ref186] Shourie A. Tomar P. Srivastava D. Chauhan R. (2014). Enhanced biosynthesis of quercetin occurs as a photoprotective measure in *Lycopersicon esculentum* mill. Under acute UV-B exposure. Braz. Arch. Biol. Technol. 57, 317–325. doi: 10.1590/S1516-8913201401678

[ref187] Shourie A. Vijayalakshmi U. (2022). Fungal diversity and its role in mycoremediation. Geomicrobiol J. 39, 426–444. doi: 10.1080/01490451.2022.2032883, PMID: 32535716

[ref188] Šimura J. Antoniadi I. Široká J. Tarkowská D. Strnad M. Ljung K.. (2018). Plant Hormonomics: multiple phytohormone profiling by targeted metabolomics. Plant Physiol. 177, 476–489. doi: 10.1104/pp.18.00293, PMID: 29703867 PMC6001343

[ref189] Singer E. Bushnell B. Coleman-Derr D. Bowman B. Bowers R. M. Levy A.. (2016). High-resolution phylogenetic microbial community profiling. ISME J. 10, 2020–2032. doi: 10.1038/ismej.2015.249, PMID: 26859772 PMC5029162

[ref190] Singh U. Khemka N. Rajkumar M. S. Garg R. Jain M. (2017). PLncPRO for prediction of long non-coding RNAs (lncRNAs) in plants and its application for discovery of abiotic stress-responsive lncRNAs in rice and chickpea. Nucleic Acids Res. 45:e183. doi: 10.1093/nar/gkx866, PMID: 29036354 PMC5727461

[ref191] Singh U. B. Malviya D. Singh S. Kumar M. Sahu P. K. Singh H. V.. (2019). *Trichoderma harzianum*- and methyl jasmonate-induced resistance to *Bipolaris sorokiniana* through enhanced phenylpropanoid activities in bread wheat (*Triticum aestivum* L.). Front. Microbiol. 10:1697. doi: 10.3389/fmicb.2019.01697 31417511 PMC6685482

[ref192] Singh N. Rai V. Singh N. K. (2020). Multi-omics strategies and prospects to enhance seed quality and nutritional traits in pigeonpea. Nucleus 63, 249–256. doi: 10.1007/s13237-020-00341-0

[ref193] Singh D. P. Singh V. Gupta V. K. Shukla R. Prabha R. Sarma B. K.. (2020). Microbial inoculation in rice regulates antioxidative reactions and defence related genes to mitigate drought stress. Sci. Rep. 10:4818. doi: 10.1038/s41598-020-61140-w 32179779 PMC7076003

[ref194] Solanki M. K. Solanki A. C. Rai S. Srivastava S. Kashyap B. K. Divvela P. K.. (2022). Functional interplay between antagonistic bacteria and *Rhizoctonia solani* in the tomato plant Rhizosphere. Front. Microbiol. 13:990850. doi: 10.3389/fmicb.2022.990850, PMID: 36225362 PMC9548980

[ref195] Solden L. Lloyd K. Wrighton K. (2016). The bright side of microbial dark matter: lessons learned from the uncultivated majority. Curr. Opin. Microbiol. 31, 217–226. doi: 10.1016/j.mib.2016.04.020, PMID: 27196505

[ref196] Sorty A. M. Meena K. K. Choudhary K. Bitla U. M. Minhas P. S. Krishnani K. K. (2016). Effect of plant growth promoting bacteria associated with halophytic weed (*Psoralea corylifolia* L.) on germination and seedling growth of wheat under saline conditions. Appl. Biochem. Biotechnol. 180, 872–882. doi: 10.1007/s12010-016-2139-z, PMID: 27215915

[ref197] Subramanian P. Mageswari A. Kim K. Lee Y. Sa T. (2015). Psychrotolerant endophytic *pseudomonas* sp. strains *OB155* and *OS261* induced chilling resistance in tomato plants (*Solanum lycopersicum* mill.) by activation of their antioxidant capacity. Mol. Plant-Microbe Interact. 28, 1073–1081. doi: 10.1094/MPMI-01-15-0021-R, PMID: 26075827

[ref198] Sultana S. Alam S. Karim M. M. (2021). Screening of siderophore-producing salt-tolerant rhizobacteria suitable for supporting plant growth in saline soils with iron limitation. J. Agric. Food Res. 4:100150. doi: 10.1016/j.jafr.2021.100150

[ref199] Sun C. Johnson J. M. Cai D. Sherameti I. Oelmüller R. Lou B. (2010). *Piriformospora indica* confers drought tolerance in Chinese cabbage leaves by stimulating antioxidant enzymes, the expression of drought-related genes and the plastid-localized CAS protein. J. Plant Physiol. 167, 1009–1017. doi: 10.1016/j.jplph.2010.02.013, PMID: 20471134

[ref200] Swenson W. Wilson D. S. Elias R. (2000). Artificial ecosystem selection. *Proc. Natl. Acad. Sci.* U. S. A. 97, 9110–9114. doi: 10.1073/pnas.150237597, PMID: 10890915 PMC16830

[ref201] Syed A. Elgorban A. M. Bahkali A. H. Eswaramoorthy R. Iqbal R. K. Danish S. (2023). Metal-tolerant and siderophore producing pseudomonas fluorescence and Trichoderma spp. improved the growth, biochemical features and yield attributes of chickpea by lowering cd uptake. Sci. Rep. 13:4471. doi: 10.1038/s41598-023-31330-3 36934106 PMC10024765

[ref202] Sziderics A. H. Rasche F. Trognitz F. Sessitsch A. Wilhelm E. (2007). Bacterial endophytes contribute to abiotic stress adaptation in pepper plants (*Capsicum annuum* L.). Canad. J. Microbiol. 53, 1195–1202. doi: 10.1139/W07-082, PMID: 18026213

[ref203] Teo H. M. Aziz A. Wahizatul A. A. Bhubalan K. Siti N. M. S. Muhamad S. C. I.. (2022). Setting a plausible route for saline soil-based crop cultivations by application of beneficial halophyte-associated bacteria: a review. Microorganisms. 10:657. doi: 10.3390/microorganisms10030657, PMID: 35336232 PMC8953261

[ref204] Timm C. M. Pelletier D. A. Jawdy S. S. Gunter L. E. Henning J. A. Engle N.. (2016). Two poplar-associated bacterial isolates induce additive favorable responses in a constructed plant-microbiome system. Front. Plant Sci. 7:497. doi: 10.3389/fpls.2016.00497, PMID: 27200001 PMC4845692

[ref205] Tittabutr P. Piromyou P. Longtonglang A. Noisa-Ngiam R. Boonkerd N. Teaumroong N. (2013). Alleviation of the effect of environmental stresses using coinoculation of mungbean by *Bradyrhizobium* and rhizobacteria containing stress induced ACC deaminase enzyme. Soil Sci. Plant Nutr. 59, 559–571. doi: 10.1080/00380768.2013.804391

[ref206] Tiwari S. Lata C. (2018). Heavy metal stress, signaling, and tolerance due to plant-associated microbes: an overview. Front. Plant Sci. 9:452. doi: 10.3389/fpls.2018.00452, PMID: 29681916 PMC5897519

[ref207] Trivedi P. Mattupalli C. Eversole K. Leach J. E. (2021). Enabling sustainable agriculture through understanding and enhancement of microbiomes. New Phytol. 230, 2129–2147. doi: 10.1111/nph.17319, PMID: 33657660

[ref208] Ul Haq S. Khan A. Ali M. Khattak A. M. Gai W. X. Zhang H. X.. (2019). Heat shock proteins: dynamic biomolecules to counter plant biotic and abiotic stresses. Int. J. Mol. Sci. 20:5321. doi: 10.3390/ijms20215321, PMID: 31731530 PMC6862505

[ref209] Uzma M. Iqbal A. Hasnain S. (2022). Drought tolerance induction and growth promotion by indole acetic acid producing *Pseudomonas aeruginosa* in *Vigna radiata*. PLoS One 17:e0262932. doi: 10.1371/journal.pone.0262932, PMID: 35120147 PMC8815908

[ref210] Varshney R. K. Hiremath P. J. Lekha P. Kashiwagi J. Balaji J. Deokar A. A.. (2009). A comprehensive resource of drought-and salinity responsive ESTs for gene discovery and marker development in chickpea (*Cicer arietinum* L.). BMC Genomics 10:523. doi: 10.1186/1471-2164-10-523, PMID: 19912666 PMC2784481

[ref211] Veeramachaneni S. Ramachandrudu K. (2020). Changes in growth, microbial and enzyme activities in oil palm nursery in response to bioinoculants and chemical fertilizers. Arch. Agron. Soil Sci. 66, 545–558. doi: 10.1080/03650340.2019.1628343

[ref212] Velásquez A. C. Castroverde C. D. M. He S. Y. (2018). Plant–pathogen warfare under changing climate conditions. Curr. Biol. 28, R619–R634. doi: 10.1016/j.cub.2018.03.054, PMID: 29787730 PMC5967643

[ref213] Veliz E. A. Martínez-Hidalgo P. Hirsch A. M. (2017). Chitinase-producing bacteria and their role in biocontrol. AIMS Microbiol. 3, 689–705. doi: 10.3934/microbiol.2017.3.689, PMID: 31294182 PMC6604996

[ref214] Vinayarani G. Prakash H. (2018). Growth promoting rhizospheric and endophytic bacteria from *Curcuma longa* L. as biocontrol agents against rhizome rot and leaf blight diseases. Plant Pathol. J. 34:218. doi: 10.5423/PPJ.OA.11.2017.0225 29887778 PMC5985648

[ref215] Wang B. Li Z. Ran Q. Li P. Peng Z. Zhang J. (2018). ZmNF-YB16 overexpression improves drought resistance and yield by enhancing photosynthesis and the antioxidant capacity of maize plants. Front. Plant Sci. 9:709. doi: 10.3389/fpls.2018.00709 29896208 PMC5986874

[ref216] Wang X. Liu Y. Jia Y. Gu H. Ma H. Yu T.. (2012). Transcriptional responses to drought stress in root and leaf of chickpea seedling. Plant Mol. Biol. Report. 39, 8147–8158. doi: 10.1007/s11033-012-1662-4, PMID: 22562393

[ref217] Wang B. Sun Y. F. Song N. Wang X. J. Feng H. Huang L. L.. (2013). Identification of UV-B-induced microRNAs in wheat. Genet. Mol. Res. 12, 4213–4221. doi: 10.4238/2013.October.7.7, PMID: 24114216

[ref218] Wang B. Zhai H. He S. Zhang H. Ren Z. Zhang D.. (2016). A vacuolar Na+/H+ antiporter gene, IbNHX2, enhances salt and drought tolerance in transgenic sweet potato. Sci. Hortic. 201, 153–166. doi: 10.1016/j.scienta.2016.01.027

[ref219] White R. A. Rivas-Ubach A. Borkum M. I. Köberl M. Bilbao A. Colby S. M.. (2017). The state of rhizospheric science in the era of multi-omics: a practical guide to omics technologies. Rhizosphere. 3, 212–221. doi: 10.1016/j.rhisph.2017.05.003

[ref220] Win K. T. Oo A. Z. Yokoyama T. (2022). Plant growth and yield response to salinity stress of Rice grown under the application of different nitrogen levels and *Bacillus pumilus* strain TUAT-1. Crops 2, 435–444. doi: 10.3390/crops2040031

[ref221] Yang J. Kloepper J. W. Ryu C. M. (2009). Rhizosphere bacteria help plants tolerate abiotic stress. Trends in plant Sci. 14, 1–4. doi: 10.1016/j.tplants.2008.10.004, PMID: 19056309

[ref222] Yasmin S. D’Souza D. (2010). Effects of pesticides on the growth and reproduction of earthworm: a review. Appl. Environ. Soil Sci. 2010:e678360. doi: 10.1155/2010/678360

[ref223] Yasmin H. Naeem S. Bakhtawar M. Jabeen Z. Nosheen A. Naz R.. (2020). Halotolerant rhizobacteria *pseudomonas pseudoalcaligenes* and *Bacillus subtilis* mediate systemic tolerance in hydroponically grown soybean (*Glycine max* L.) against salinity stress. PLoS One 15:e0231348. doi: 10.1371/journal.pone.0231348, PMID: 32298338 PMC7162512

[ref224] Zahoor M. Irshad M. Rahman H. Qasim M. Afridi S. G. Qadir M.. (2017). Alleviation of heavy metal toxicity and phytostimulation of *Brassica campestris* L. by endophytic *Mucor* sp. *MHR-7*. Ecotoxicol. Environ. Saf. 142, 139–149. doi: 10.1016/j.ecoenv.2017.04.005, PMID: 28407499

[ref225] Zainab N. Khan A. A. Azeem M. A. Ali B. Wang T. Shi F.. (2021). PGPR-mediated plant growth attributes and metal extraction ability of *Sesbania sesban* L. in industrially contaminated soils. Agronomy 11:1820. doi: 10.3390/agronomy11091820

[ref226] Zandalinas S. I. Mittler R. (2022). Plant responses to multifactorial stress combination. New Phytol. 234, 1161–1167. doi: 10.1111/nph.18087, PMID: 35278228

[ref227] Zandi P. Schnug E. (2022). Reactive oxygen species, antioxidant responses and implications from a microbial modulation perspective. Biology (Basel) 11:155. doi: 10.3390/biology11020155, PMID: 35205022 PMC8869449

[ref228] Zenda T. Liu S. Dong A. Li J. Wang Y. Liu X.. (2021). Omics-facilitated crop improvement for climate resilience and superior nutritive value. Front. Plant Sci. 12:774994. doi: 10.3389/fpls.2021.774994, PMID: 34925418 PMC8672198

[ref229] Zhang B. Wang Q. (2015). MicroRNA-based biotechnology for plant improvement. J. Cell. Physiol. 230, 1–15. doi: 10.1002/jcp.24685, PMID: 24909308

[ref230] Zhang Z. Wei L. Zou X. Tao Y. Liu Z. Zheng Y. (2008). Submergence-responsive MicroRNAs are potentially involved in the regulation of morphological and metabolic adaptations in maize root cells. Ann. Bot. 102, 509–519. doi: 10.1093/aob/mcn129, PMID: 18669574 PMC2701776

[ref231] Zhang F. Yang J. Zhang N. Wu J. Si H. (2022). Roles of microRNAs in abiotic stress response and characteristics regulation of plant. Front. Plant Sci. 13:919243. doi: 10.3389/fpls.2022.919243, PMID: 36092392 PMC9459240

[ref232] Zhang Y. Zhu X. Chen X. Song C. Zou Z. Wang Y.. (2014). Identification and characterization of cold-responsive microRNAs in tea plant (*Camellia sinensis*) and their targets using high-throughput sequencing and degradome analysis. BMC Plant Biol. 14:271. doi: 10.1186/s12870-014-0271-x 25330732 PMC4209041

[ref233] Zhang H. Zhu J. Gong Z. Zhu J. K. (2022). Abiotic stress responses in plants. Nat. Rev. Genet. 23, 104–119. doi: 10.1038/s41576-021-00413-0, PMID: 34561623

[ref234] Zhao C. Liu B. Piao S. Wang X. Lobell D. B. Huang Y.. (2017). Temperature increase reduces global yields of major crops in four independent estimates. Proc. Natl. Acad. Sci. 114, 9326–9331. doi: 10.1073/pnas.1701762114, PMID: 28811375 PMC5584412

[ref235] Zhou L. Liu Y. Liu Z. Kong D. Duan M. Luo L. (2010). Genome-wide identification and analysis of drought-responsive microRNAs in *Oryza sativa*. J. Exp. Bot. 61, 4157–4168. doi: 10.1093/jxb/erq237, PMID: 20729483

[ref236] Zong N. Li X. Wang L. Wang Y. Wen H. Li L.. (2018). Maize ABP2 enhances tolerance to drought and salt stress in transgenic *Arabidopsis*. J. Int. Agric. 17, 2379–2393. doi: 10.1016/S2095-3119(18)61947-61941

